# Potassium‐Doped Borophane Nanosheets: A Multifunctional Platform for Reversible Hydrogen Storage and Metal‐Free Hydrogen Transfer

**DOI:** 10.1002/smll.202511090

**Published:** 2026-02-23

**Authors:** Rajamohanan Sobhana Anju, Pankaj Kumar, Dhanaji R. Naikwadi, Bettina Baumgartner, Savi Chaudhary, Atul Bansode, Merel C. Konings, Freek Ariese, Erdni D. Batyrev, Prasad Gonugunta, Vimal Chandra Srivastava, Ramaswamy Murugavel, N. Raveendran Shiju

**Affiliations:** ^1^ Catalysis Engineering Group, Van ‘t Hoff Institute for Molecular Sciences University of Amsterdam Amsterdam 1090GD The Netherlands; ^2^ Department of Chemical Engineering Indian Institute of Technology Roorkee Roorkee India; ^3^ Department of Chemical Engineering Delft University of Technology Delft The Netherlands; ^4^ Homogeneous, Supramolecular and Bio‐Inspired Catalysis group Van't Hoff Institute for Molecular Sciences University of Amsterdam Amsterdam The Netherlands; ^5^ Department of Chemistry Indian Institute of Technology Bombay Mumbai India; ^6^ LaserLaB Department of Physics and Astronomy Vrije Universiteit Amsterdam Amsterdam The Netherlands; ^7^ Tata Steel Research & Development IJmuiden The Netherlands; ^8^ Department of Material Science and Engineering Delft University of Technology Delft The Netherlands

**Keywords:** 2D boron, biomass conversion, borophane, borophene, catalysis, hydrogen storage, hydrogen transfer, levulinic acid reduction, potassium doped, γ‐valerolactone

## Abstract

2D borophene has long been proposed as a promising hydrogen storage material, but experimental demonstrations remain limited to boron hydride sheets derived from MgB_2_. Here, we report the synthesis of potassium‐doped borophane (BH) nanosheets, which serve as a high‐capacity, reversible hydrogen storage platform and metal‐free reducing agent. Through selective hydride transfer, the BH sheet efficiently converted levulinic acid (LA) to γ‐valerolactone (GVL) under mild reaction conditions. Density functional theory (DFT) predicts a theoretical hydrogen content of 4.2 wt.% for the potassium‐doped BH sheet. Remarkably, the dehydrogenated BH sheets can be partially regenerated under 50 bar H_2_, demonstrating reversible hydrogen storage. This work serves as an experimental validation for alkali‐metal‐modified borophanes acting as a multifunctional material for hydrogen storage and transfer, opening avenues for sustainable energy and other applications.

## Graphical abstract

Borophane in dual roles: as a reductant in the conversion of levulinic acid to γ‐valerolactone and as a reversible hydrogen reservoir through partial rehydrogenation.

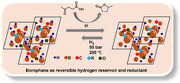

## 1 Introduction

The world is transitioning toward renewable energy sources, with hydrogen emerging as a promising energy carrier [1]. While molecular hydrogen boasts a high energy density by weight, its practical storage in compressed or liquefied forms faces significant challenges concerning safety, infrastructure, and energy efficiency [2]. Thus, achieving efficient and reversible hydrogen storage remains a major hurdle. Among the two primary hydrogen storage approaches, chemical storage (materials‐based) offers a safer and more compact alternative to conventional physical storage techniques (compressed or liquefied hydrogen). Traditional storage materials, including borohydrides, metal hydrides, and porous frameworks, not only serve as hydrogen carriers but also act as powerful reducing agents in key organic transformations. However, they are often affected by issues like poor reversibility, unfavourable thermodynamics due to high reaction enthalpy, and elevated dehydrogenation temperatures [3, 4]. Consequently, substantial efforts have been devoted to developing materials and strategies that lower the thermodynamic and kinetic barriers to hydrogen uptake and release, including solar‐driven reversible hydrogen storage that enables hydride hydrogenation/dehydrogenation under milder conditions with reduced external energy input [5, 6, 7, 8, 9].

In recent years, 2D materials have garnered significant attention as lightweight platforms for hydrogen storage and as highly active catalysts for hydrogen uptake and release, owing to their distinctive electronic structures and tunable surface chemistry [9, 10, 11, 12, 13, 14, 15, 16, 17, 18, 19]. Borophene, a 2D allotrope of boron, is particularly effective at promoting strong interactions with hydrogen due to its tunable morphology and metallic conductivity [20, 21, 22, 23]. A recent computational study, for instance, demonstrated that borophene possesses hydrogen storage capacities approaching the US Department of Energy's (DOE) target of 5.5 wt.% [24]. Furthermore, numerous computational analyses have explored the hydrogen storage potential of pristine borophenes, those with defects, and borophenes modified with alkali or transition metals, as well as heteroatoms [12, 25, 26, 27, 28]. While pristine borophenes exhibit limited ability to bind hydrogen, the findings consistently indicate that both defective and modified borophenes offer promising capabilities, marked by significant binding energies and gravimetric densities. The adsorption of hydrogen on the surface of defective or modified borophene results in charge transfer and bond formation, thereby altering the electronic and structural characteristics of the material.

Conversely, hydrogen boride sheets and borophanes, which are hydrogenated or reduced forms of borophene, integrate hydrogens directly into their structure, forming stable B─H bonds as an intrinsic part of the material [29, 30, 31]. Photoirradiation of hydrogen boride sheets at ambient temperature and pressure has been shown to trigger the release of up to 8 wt.% hydrogen, a storage capacity comparable to that of several metal‐based hydrogen storage materials reported in earlier research [23, 32]. Notably, borophanes modified with alkali metals can achieve high hydrogen storage capacities of up to 11.5 wt.%, enabling controlled hydrogen uptake and release under moderate conditions [20]. This stands in stark contrast to conventional materials such as metals and complex hydrides, which often suffer from strong hydrogen binding, leading to irreversibility, slow kinetics, and high dehydrogenation temperatures, thus limiting their practical utility.

In this report, we demonstrate that alkali metal‐modified borophane (BH) sheets serve as chemoselective reducing agents and reversible hydrogen storage media under mild conditions. Using the hydrogenation of levulinic acid (LA) to γ‐valerolactone (GVL) as a model reaction, we establish that these BH sheets can deliver hydride equivalents from surface‐bound B─H species with high efficiency and selectivity, without the need for molecular hydrogen, high temperatures, and pressures, or precious metal catalysts. A series of BH sheets was synthesized by varying the molar ratio of NaBH_4_ to HCOOK from 9:1 to 2:1, designated as BH(9:1) through BH(2:1). Notably, BH(6:1) exhibited superior performance toward LA reduction. The study presents a comprehensive investigation covering the synthesis, characterization, redox behaviour, and mechanistic analysis of BH sheets doped with potassium. To rationalize the experimental observations, we employed density functional theory (DFT) and explored the electronic structure of the BH sheet following the methodology reported by Kumar et al. [33, 34]. Computational details are provided in the Supporting Information.

**FIGURE 1:**
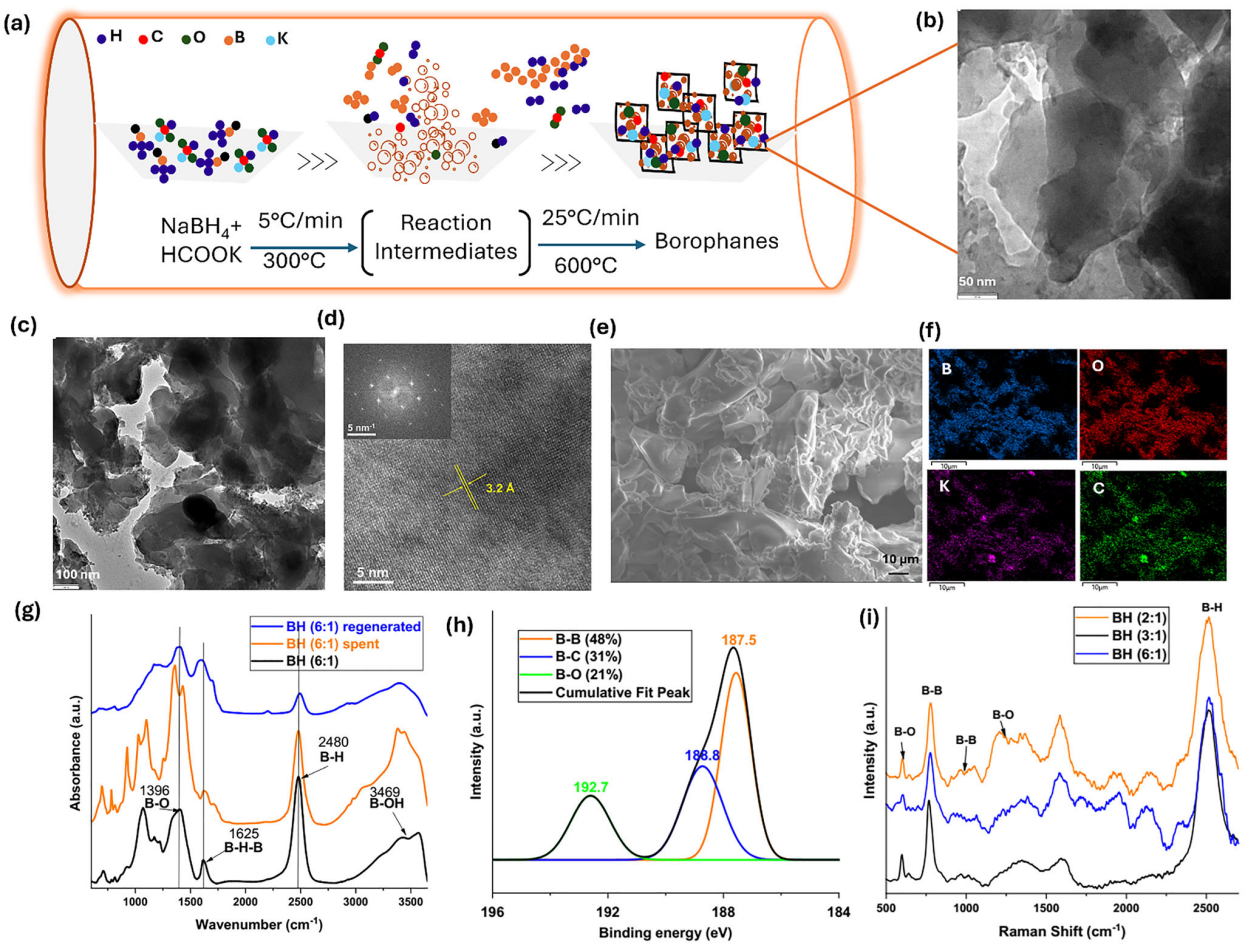
Synthesis and characterisation of BHs. (a) Schematic representation for the synthesis of BHs; (b,c) TEM image of BH(6:1); (d) HR‐TEM image of BH(6:1); (e) SEM image of BH(6:1); (f) SEM‐EDS analysis of BH(6:1); (g) FTIR spectra of pristine BH(6:1), spent BH(6:1) and regenerated BH(6:1); (h) B 1s XPS analysis of BH(6:1) and (i) Raman spectra of BH sheets.

The BH sheets were synthesized in a tubular furnace using a stepwise thermal decomposition method involving sodium borohydride (NaBH_4_) and potassium formate (HCOOK) (Figure 1a; Scheme S1) [35]. During the synthesis, decomposition of potassium formate generates a reducing (hydrogen‐rich) atmosphere. In the presence of this, the unstable boron intermediates formed from the thermal decomposition of NaBH_4_ stabilise to yield Borophanes (BH), a hydrogenated boron‐rich lattice [35].

State‐of‐the‐art characterizations together with DFT revealed the structure of the BH lattice. Scanning electron microscopy (SEM) (Figure 1e; Figure S1) and transmission electron microscopy (TEM) images (Figure 1b–d; Figure S2) of the pristine BH revealed a crumpled 2D sheet‐like morphology with a large external surface and many exposed sheet edges. Analysis of the textural characteristics (Figure S3) indicated a minimal surface area (1.21 m^2^/g) for the pristine BH(6:1) sheet. Energy‐dispersive X‐ray spectroscopy (EDS) (Figure 1f; Figure S1e) confirmed the presence and distribution of B, O, C, Na, and K across the BH sheet. Furthermore, Fourier‐Transform‐infrared (FT‐IR, Figure 1g, black) and Raman (Figure 1i) spectroscopy confirmed the reduced nature of the BH lattice [29, 30, 35]. Characteristic bands corresponding to B─H─B bridging (1625 cm^−^^1^ in the FT‐IR spectrum), B─H terminal bonds (2480 cm^−^^1^ in FT‐IR and 2500 cm^−^^1^ in Raman), and B─O motifs were observed [36]. The coexistence of hydride‐like B─H species and protic O─H groups on the same lattice provides both hydrogen and electron density that can be transferred to an organic substrate. These surface functionalities thus serve as the molecular origin of BH's reducibility. The sharp band at 760 cm^−1^ and the broad band at 1049 cm^−1^ in the Raman spectra of BH sheets represent the Eg and A1g+Eg vibrational modes of the B─B bonds. The chemical composition of the BH surface was further examined using X‐ray photoelectron spectroscopy (XPS), revealing a lattice rich in B─B bonds, along with some B─C and B─O bonds (Figure 1h). The significant fraction of low‐binding‐energy B species demonstrates that the majority of the lattice is in a reduced state, capable of donating electrons during reaction. The K 2p XPS spectrum exhibits a single K^+^ doublet (K 2p_3/2_ at 293.3 eV; K 2p_1/2_ at 296.0 eV; ΔSO ≈ 2.7 eV), confirming that potassium is present predominantly in an ionic state on the BH sheet surface (Figure S4). Cationic K is consistent with charge transfer/promoter effects that polarize the B─H framework. This strengthens the hydride‐driven reducing character and modulates H binding/activation pathways relevant to reversible hydrogen uptake and release. Mulliken charge analysis via DFT calculations also showed the cationic properties of K, with a Mulliken charge of 0.728 in the optimized structure.

**FIGURE 2:**
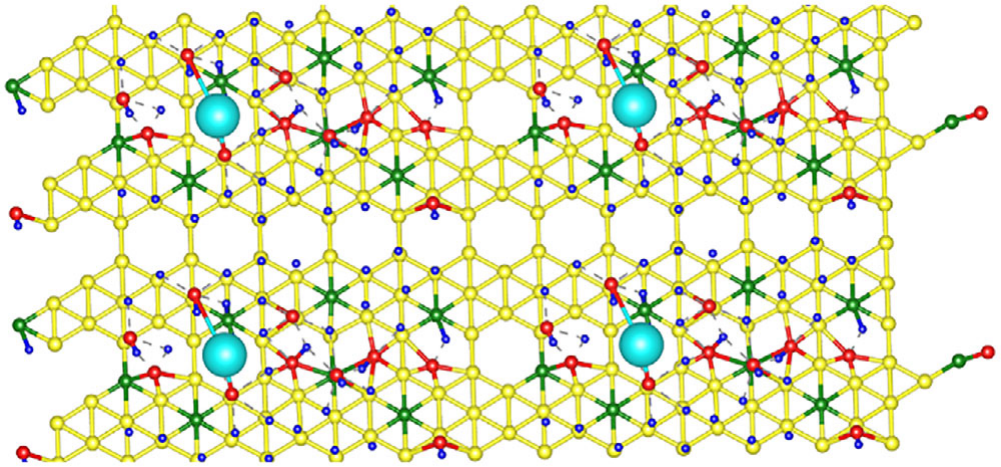
DFT optimised β_12_ phase of BH (6:1) (Boron in yellow; Carbon in green; Oxygen in red; Hydrogen in blue and Potassium in cyan).

To elucidate the atomic‐scale structure and interactions, Striped, β_12_, and X_3_ phases of a pure borophene monolayer (Figure 2; Figure S17) were employed as reference structures for molecular simulations. For systems containing B, O, C, K, and H atoms, the β_12_ phase exhibited the lowest total energy (−1308.143 Ry), followed by X_3_ (−1307.680 Ry) and Striped (−1307.546 Ry), indicating its thermodynamic stability. In contrast, for pure borophene comprising only B, H, and O atoms, the Striped phase was marginally more stable (−1144.885 Ry) than X_3_ (−1144.768 Ry) and β_12_ (−1144.617 Ry).

Mulliken population analysis of optimized Gaussian structures showed average charges of −0.545 for C atoms and +0.728 for K atoms, while B and H atoms exhibited both positive and negative charges depending on the local coordination environment. Molecular dynamics (MD) simulations in the N–V–T ensemble, employing the Anderson thermostat at 250 and 300 K, were conducted to evaluate H_2_ storage content and molecular mobility (Figures S18–S22). The simulations indicate that the structure is initially stable, followed by stepwise H_2_ evolution predominantly from boron sites, with no hydrogen desorption from carbon sites. Moreover, increasing the temperature from 250 to 300 K did not lead to any measurable increase in the number of H_2_ molecules released from the surface.

Insights from structural characterization and DFT calculations indicated that the BH lattice can host several hydride‐like hydrogen atoms. These hydrogen species are weakly bound and possess sufficient reactivity to participate in redox transformations, suggesting that the BH sheet could serve as a readily available source of hydrogen. This is significant, as it positions BH sheets as a promising candidate for chemical hydrogen storage systems. Moreover, interestingly, the thermogravimetric analysis (TGA) of BH(6:1) reveals a substantial mass loss (∼18%) between 60°C and 180°C, indicating the release of kinetically labile hydrogen at mild temperatures, a key feature for hydrogen storage (Figure S5a). While ammonia boranes exhibit similar thermal behaviour between temperatures from 80°C to 200°C, their decomposition generates unwanted by‐products such as borazine, aminoboranes, and ammonia [37]. In contrast, BH(6:1) released only H_2_ (as confirmed by GC‐TCD analysis of the gases evolved between 30°C–300°C), promising its potential as a clean hydrogen storage material (Figure S5b,c). The higher experimental H_2_ release, compared to theoretical hydrogen content, indicates that the synthesized BH material is non‐stoichiometric and hydrogen‐rich (BH_x_) relative to the idealized model composition used for the DFT calculations.

To probe the intrinsic reducing ability and hydrogen release behaviour of the BH sheets, we employed LA, a readily available biomass‐derived platform chemical, as a model substrate [38, 39, 40, 41, 42, 43]. Remarkably, under mild conditions and without any other external hydrogen source, BH sheets selectively reduced LA to GVL, a key molecule in the circular bioeconomy, functioning as a green solvent, a sustainable fuel additive, and a precursor to a variety of value‐added chemicals, highlighting the dual functionality of BH as both a hydrogen reservoir and a metal‐free reducing agent. DFT calculations identified potassium in the β_12_ lattice as essential for LA adsorption (E_ads = 0.0347 Ry, ≈0.472 eV). LA binding reduced the Mulliken charge on K to 0.540 from the original value of 0.728, evidencing substantial charge transfer to the adsorbate‐substrate complex. On the other hand, potassium removal abolished LA adsorption, confirming its pivotal electronic and structural role. The adsorption study was also performed by replacing K with a Na atom. It was found that adsorption required E_ads = 6.82568 Ry, ≈ 92.8682 eV. This enormous energy confirms that BH with Na is incapable of LA adsorption.

Among the series of BH sheets synthesized, BH(6:1) exhibited outstanding performance, achieving quantitative conversion and 100% selectivity for GVL. In contrast to conventional hydrogenation strategies that rely on high‐pressure H_2_, elevated temperatures, and precious metal catalysts (e.g., Ru, Pd, Pt), the BH‐mediated process proceeds under significantly milder conditions, delivering a single desirable product with exceptional chemo selectivity [38, 39, 40].

We then performed a solvent scope study with a range of solvents of varying polarity and proton‐donating ability characteristics (Table 1) to better understand the role of the reaction medium. Interestingly, complete conversion was observed only in non‐polar aprotic solvents such as toluene. The reaction still proceeded in polar aprotic solvents but with significantly reduced efficiency; conversion dropped nearly threefold. Notably, no reaction occurred in polar protic solvents (e.g., ethanol, water), a finding that contrasted with conventional reduction reactions involving classical metal hydride systems or analogous reducing agents, where protic solvents are often necessary for proton shuttling [44, 45]. The observation that BH remained reactive in non‐polar aprotic solvents likely indicates that its surface hydride‐like species are stable and accessible. In contrast, the diminished activity in polar aprotic solvents suggests possible deactivation/blocking of these sites through dipolar or Lewis basic interactions.

**TABLE 1:** (a) Reducing property of the different BH sheets and (b) solvent scope for the reaction.

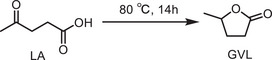			
Reducing property of different BH sheets			
Entry	BH used	Conversion of LA (%)	Yield (%)
1	BH(9:1)	68	61
2	BH(6:1)	100	95
3	BH(3:1)	91	85
4	BH(2:1)	63	58

(b) Solvent scope of the reaction			
Entry	Solvent	Nature of the solvent	Conversion of LA (%)
1	Water	Polar protic	nil
2	Isopropanol^a^	Polar protic	nil
3	Ethanol^a^	Polar protic	nil
4	Formic Acid	Polar protic	nil
5	Ethyl acetate^a^	Polar aprotic	10
6	Acetonitrile	Polar aprotic	24
7	DMSO	Polar aprotic	26
8	Toluene	Nonpolar aprotic	99
9	Octane	Nonpolar aprotic	95
10	Benzene^a^	Nonpolar aprotic	89

^a^ reaction temperature is 75°C, instead of 80°C.

The pronounced suppression of reactivity in protic solvents indicated a mechanistically distinct reduction pathway. To examine this, we conducted in situ and ex situ FT‐IR analyses. Comparative ex situ FT‐IR spectra of BH(6:1) before and after reaction with LA in toluene at room temperature, followed by filtration and air drying, revealed distinct new bands (Figure S6a), consistent with LA adsorption on the BH(6:1) surface. This highlights effective substrate‐surface interactions in nonpolar aprotic media. In contrast, reactions in ethanol showed no evidence of LA adsorption, including the absence of the characteristic ketone band (∼1718 cm^−^^1^), and displayed only the spectral features of pristine BH(6:1) (Figure S6a). These findings suggest that polar protic solvents disrupt LA adsorption on BH(6:1), likely via solvation or competitive hydrogen bonding, thereby impeding the initial substrate‐surface association essential for reactivity.

**FIGURE 3:**
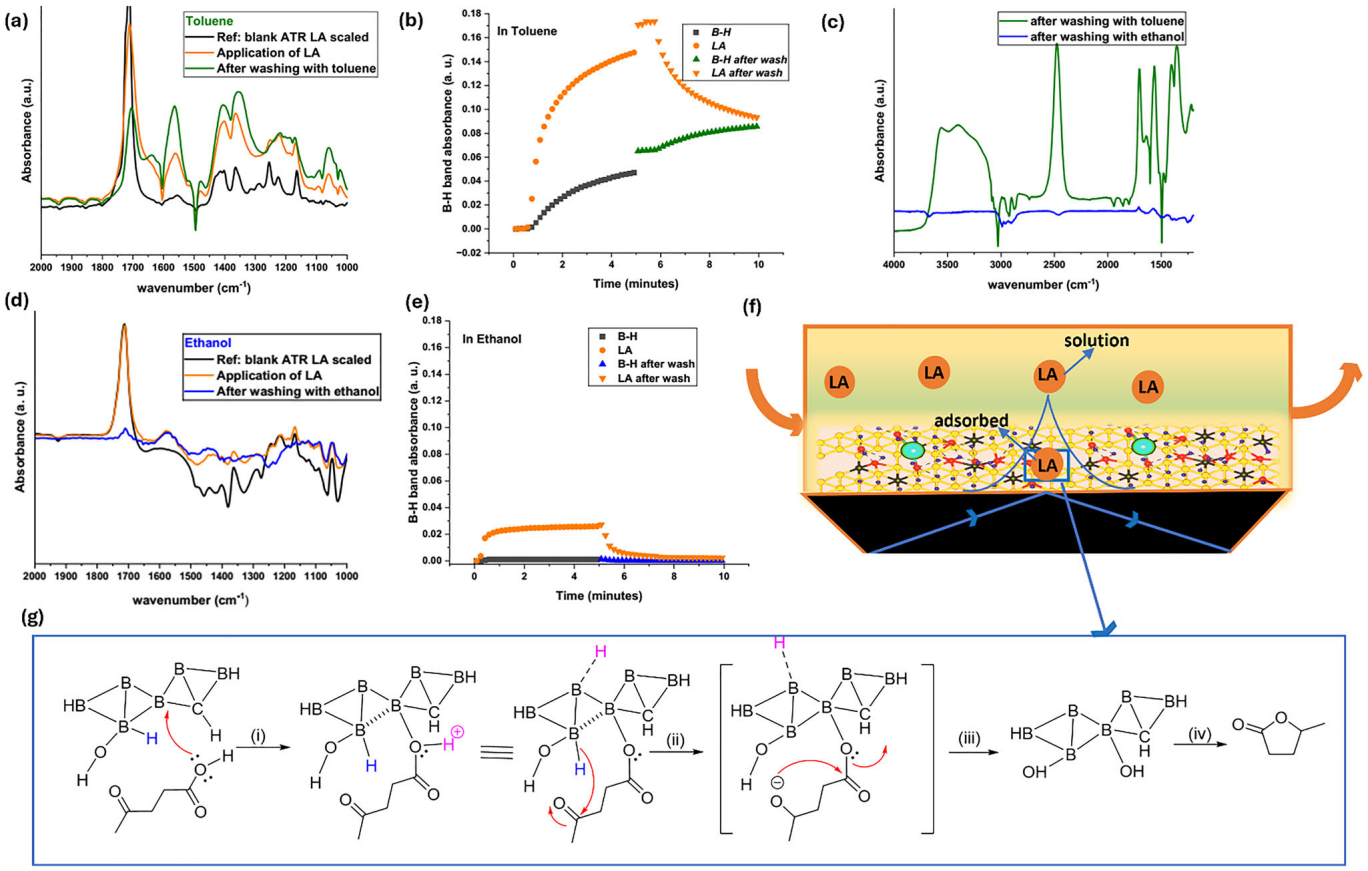
(a) In situ Fourier transform‐infrared (FT‐IR) attenuated total reflectance (ATR) spectra during the adsorption of LA using BH(6:1) in toluene; (b) corresponding absorbance Vs. time profile; (c) Comparison of the FT‐IR spectra recorded after LA adsorption on BH(6:1) in toluene and ethanol; (d) In situ Fourier transform‐infrared (FT‐IR) attenuated total reflectance (ATR) spectra during the reduction of LA using BH(6:1) in ethanol; (e) corresponding absorbance Vs time profile; (f) Schematic representation of the FTIR‐ATR spectroscopic set up with total reflection of the IR beam that generates the evanescent field in which absorption takes place. Note that the evanescent field stretched beyond the BH deposited on the surface into the solute phase above; (g) Proposed reaction mechanism based on insights from in situ FTIR observations.

To further prove this hypothesis, we performed in situ FT‐IR spectroscopy in attenuated total reflection (ATR) configuration using a BH drop‐casted on silicon (Si) ATR crystals and placed into a custom flow cell (Figure 3a–f, see Supporting Information for experimental details and Figure 3f for schematics of the setup). The respective solvent was flushed until a stable baseline was reached. Subsequently, the adsorption and desorption of LA dissolved in either ethanol or toluene for 5 min each were monitored by recording an IR spectrum every 10 s [46, 47]. As we probed dissolved LA in the respective solvent as well as adsorbed species within the evanescent wave during the measurements, pure solvent was flushed after the adsorption process for 5 min to differentiate dissolved and surface‐adsorbed LA. Thereby, we assessed LA bound to the surface of the catalyst and hence LA's surface affinity, indicated by the “washed” spectra in Figure 3. Note that, during the first 5 min of LA application, the spectrum shows contributions from both adsorbed LA on the catalyst and LA in solution. In toluene, the spectra displayed pronounced changes characteristic of substrate interaction and subsequent reaction (Figure 3a), whereas in ethanol, insignificant spectral changes were observed (Figure 3c,d), further indicating that LA does not interact with BH in polar protic media.

Initially, we posited that reduction might involve either terminal (B─H) or bridging (B─H–B) hydrogens [30]. However, the bridging B─H─B stretch at 1625 cm^−^^1^ remained unchanged, indicating it is not directly involved in substrate activation (Figure 3). In situ monitoring in toluene revealed a continuous increase in the terminal B─H stretching band (2480 cm^−^^1^) upon LA introduction (Figure 3b, grey). Rather than diminishing as expected for hydride transfer, the enhanced B─H signal suggested a more intricate mechanism. A plausible reaction mechanism based on these observations is illustrated in Figure 3g. The reaction began with LA adsorbing onto an electron‐deficient boron of BH(6:1) through its carboxyl group. The bands at 1350–1400 cm^−^^1^ ν_s_(COO^−^) and 1560 cm^−^^1^ ν_as_(COO^−^) upon LA application, followed by washing with toluene, supported this (Figure 3a green) [48]. This interaction polarizes the O─H bond of the carboxylic group, rendering the proton (H^+^, shown in pink) labile. The proton subsequently transfers to a neighboring electron‐deficient boron site, where it is stabilized through electron delocalization. This mechanistic step aligns with the enhanced B─H band intensity observed in the in situ FTIR spectra upon LA coordination, yielding carboxylate bands. Next, the terminal B─H hydrogen nucleophilically attacks the carbonyl carbon, effecting reduction of the carbonyl group. DFT calculations revealed that boron(s) in proximity to O and C atoms showed enhanced electropositive character, with a Mulliken charge of 0.454 vs. 0.160 in distant regions. This hints at the specific active B─H sites for LA reduction. Subsequent internal rearrangements yield GVL and a spent BH(6:1) surface.

In contrast, in ethanol (and other polar protic solvents), ethanol preferentially adsorbs onto the BH surface through hydrogen bonding or dipolar interactions with surface ─OH or B─H groups (Figure 3d,e). This competitive adsorption effectively blocks LA from accessing active sites, thereby inhibiting the reaction. Notably, ethyl and butyl levulinates failed to undergo reduction in the presence of BH sheets, underscoring the critical role of a free carboxylic acid group in facilitating the proposed mechanism (Table S1). Under analogous conditions, pyruvic acid, with a free carboxylic ‐OH group, is reduced to lactic acid, further supporting this mechanistic rationale (Table S1).

Further ex situ analysis of FT‐IR spectra showed that the BH sheets revealed a sharper, well‐defined B─H terminal stretching band for BH(6:1) relative to other BH configurations, indicative of greater structural homogeneity and a greater abundance of terminal B─H species, which are the catalytically active sites for LA reduction (Figure S6b). In contrast, BH sheets synthesized from NaBH_4_ under a H_2_ atmosphere (without HCOOK) displayed FT‐IR spectra consistent with both bridging and terminal hydrogen; yet exhibited no detectable reducing activity (Figure S6b). This underscored the critical role of potassium in activating terminal B─H bonds for hydride transfer via charge distribution within the BH lattice. Calculation of the FUKUI function performed using Gaussian and FUKUI‐Function V2 software (Figure S23) showed that atoms 1 to 10, 11 to 14, and 15 to 19 represent the activity of B, C, and O atoms, respectively. The highest activity is observed over B4, B5, B6, C13, and C14, which represent the atoms nearest to the potassium atom. C13 and C14 are directly attached to the K atom (atom 20) in the optimized structure. The highly concentrated FUKUI indices near the K atom emphasize the need for the K atom for the activity of BH. DFT calculations revealed that K incorporation enhanced the electronegativity of adjacent O atoms (Mulliken charge −0.722) and the electropositivity of neighbouring H atoms (+0.400), generating a strong localized dipole that facilitated B─H bond activation.

**FIGURE 4:**
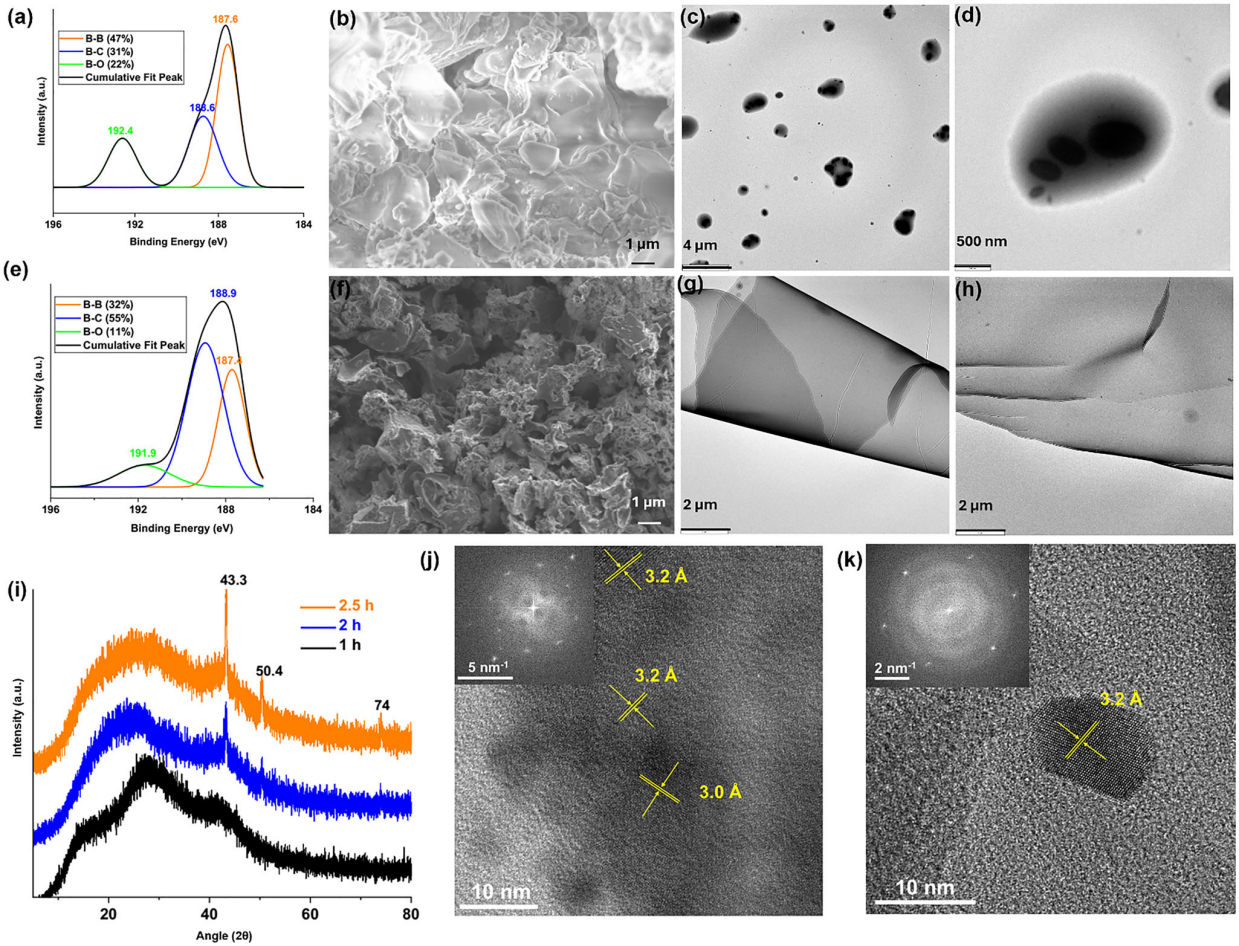
(a) B 1s XPS spectrum of spent BH(6:1); (b)SEM image of spent BH(6:1); (c,d) TEM images of spent BH(6:1); (e) B 1s XPS spectrum of regenerated BH(6:1); (f) SEM image of regenerated BH(6:1); (g,h) TEM images of regenerated BH(6:1); (i) XRD of BH(6:1) after 1–2.5 h of high pressure hydrogenation; (j) HR‐TEM image of spent BH(6:1); (k) HR‐TEM image of regenerated BH(6:1).

The spent BH sheets, recovered by simple filtration and drying after LA reduction, showed no further reactivity, indicating irreversible consumption of active hydride species under the reaction conditions. However, FT‐IR analysis (Figure 1g) revealed that only a fraction of terminal hydrogens (B─H) was used for LA reduction, suggesting partial utilization of the hydride reservoir and supporting a site‐selective reduction mechanism. As indicated in Figure 3g, assuming the consumption of one hydride equivalent from BH(6:1) per molecule of LA is reduced, only ∼26% of the lattice hydrogen is utilized during the reaction. This agrees with DFT predictions that only hydrogens bound to specific boron sites are catalytically active. B 1s XPS analysis of the spent BH(6:1) confirmed the preservation of its core surface chemistry with no significant shifts in the peaks (Figure 4a). SEM imaging revealed significant restacking and aggregation of BH sheets, and the EDS mapping suggested a boron‐rich lattice consistent with that of pristine BH(6:1) (Figure 4b; Figures S7 and S8).

Further, efforts to regenerate the hydrogen content of spent BH sheets were carried out by subjecting the material to hydrogenation at 200°C under 50 bar H_2_ pressure. Following this, the material was used for LA reduction, resulting in 33% conversion of LA to GVL, suggesting partial re‐hydrogenation (Figure S9). Analyzing the XPS spectra of regenerated BH sheets revealed that ∼15% of the B─B bonds on the lattice are irreversibly lost, likely due to bond rearrangement and/or byproduct formation. (Compare Figure 1h and Figure 4e). This is also evident from the diminished B─H band intensity observed in the FT‐IR spectra of regenerated BH(6:1) (Figure 1g). The FTIR spectrum of regenerated BH(6:1) exhibited a markedly lower B─H band intensity compared to the spent BH(6:1), while the B─H─B band intensity is substantially enhanced. These results suggest that high‐pressure hydrogenation either restores both terminal (B─H) and bridging (B─H─B) hydride species or promotes partial conversion of terminal B─H bonds into bridging B─H─B configurations under reaction conditions. Additionally, the regeneration experiments indicated that BH sheets decompose when the pressure exceeds 80 bars. Even at 50 bars, the sheets gradually degraded over time. Powder XRD analysis of regenerated BH sheets at 50 bars for varying reaction times revealed the emergence of new sharp peaks over time (Figure 4i). This, together with the reduced B─B bond content in the regenerated BH(6:1), implied the release of boron‐containing species from the BH sheet upon regeneration.

Inductively Coupled Plasma‐Optical Emission Spectrometer (ICP‐OES), XPS, and SEM‐EDS analysis consistently revealed a lower boron content in the regenerated BH(6:1) lattice compared to both pristine and spent ones (Table 2; Table S2 and, Figures S1e, S8b, S10, and S13). The K 2p XPS spectra showed no changes in peak position for the spent and regenerated BH sheets, indicating that the chemical environment of K remains essentially unchanged throughout reaction and regeneration. (Figure S11). Collectively, these observations indicate that the partial loss of catalytic activity upon regeneration originates from irreversible depletion of lattice B–B motifs, caused by pressure‐induced bond reorganization (B─H to B─H–B) and progressive boron leaching/byproduct formation under high‐pressure H_2_, which diminishes the population of active hydride‐bearing sites.

**TABLE 2:** Comparison of boron content in BH(6:1), spent BH(6:1), and regenerated BH(6:1) determined by independent analyses (SEM‐EDS, XPS, and ICP), highlighting the progressive boron depletion upon reaction and regeneration.

Name	Boron content		
	ICP (weight %)	SEM‐EDS (atomic %)	XPS (atomic %)
BH	24.8	41.8	33.2
Spent BH(6:1)	22.2	40.0	29.9
Regenerated BH(6:1)	19.5	29.6	26.1

Comparative SEM and HR‐TEM analyses of pristine, spent, and regenerated BH(6:1) revealed a striking morphological evolution: from thin sheets in the pristine BH to aggregated, globular structures in the spent state, and back to very thin sheets upon regeneration. This morphological trend is paralleled by structural transitions observed in corresponding SAED patterns, which show a shift from a well‐defined hexagonal lattice in the pristine material to a distorted hexagonal lattice in the spent state; fully restored after re‐hydrogenation (Figure 1d and Figure 4j,k). The globular morphology observed in spent BH(6:1) (Figure 4c,d; Figure S14) is likely a result of local lattice restructuring during hydrogen extraction. Surface‐localized hydrogen loss may induce lattice contraction or relaxation, generating phase‐segregated domains enriched in boron. Alternatively, localized condensation of B or BH fragments during incomplete lattice recombination could account for this morphology. Interestingly, SEM and TEM images of regenerated BH(6:1) (Figure 4g,h, Figures S12 and S15) display thinner sheet‐like structures, suggesting that high‐pressure hydrogenation promotes exfoliation of the BH lattice [49, 50].

Despite these significant morphological and structural changes, HR‐TEM images of pristine, spent, and regenerated BH(6:1) consistently revealed areas with a lattice spacing of 3.2 Å, confirming the preservation of structural integrity throughout the reversible hydrogen storage cycle. It is notable that the spent BH(6:1) also displayed an additional lattice fringe at 3.0 Å, indicative of structural domains formed upon hydrogen elimination during reduction (Figure 4j). While a major portion of the spent and regenerated materials showed an amorphous nature, these findings collectively provide compelling evidence for the transformations during the entire reversible hydrogen storage process.

These structural changes markedly compromised the cycling stability of the BH sheets. A subsequent third cycle using the BH sheets converted only ∼2%–3% of LA, indicating a severe loss of reducing capacity. This result further indicates that high‐pressure hydrogenation may not be the optimal regeneration strategy for BH sheets.

## 2 Conclusion

This study highlights the promising hydrogen‐storage potential and site‐specific reducing nature of potassium‐doped borophane sheets. The BH lattice hosts distinct terminal B─H and bridging B─H–B motifs and releases hydrogen cleanly at mild temperatures, while simultaneously enabling highly chemoselective LA to GVL conversion in aprotic, nonpolar solvents. Comprehensive structural and spectroscopic analyses reveal that only specific B─H species are involved in the reduction process, and the BH sheet largely preserves its sheet‐like morphology after partial re‐hydrogenation. Potassium is essential for substrate adsorption and for polarizing/activating the reactive B─H environment. Partial re‐hydrogenation is achieved via high‐pressure hydrogenation, though the process is limited by irreversible loss of B─B bonds and boron content during cycling. Efforts to develop more efficient regeneration processes are ongoing. In short, the findings mark a significant experimental advancement toward the development of safe, efficient, and high‐capacity hydrogen storage materials, offering a viable metal‐free platform for sustainable energy applications.

## Author Contributions

Methodology and experiment design: RSA, NRS, Investigation: RSA, DRN, BB, SC, AB, MCK, FA, EDB, PG, RM; Formal Analysis, DFT Calculations: PK; Supervision: VCS, NRS, writing – original draft: RSA, PK; Writing – review and editing: RSA, PK, DRN, BB, FA, PG, AB, VCS, RM, NRS. The authors PK, DRN and BB contributed equally to the manuscript.

## Conflicts of Interest

The authors declare no conflicts of interest.

## Supporting information




**Supporting File**: smll72884‐sup‐0001‐SuppMat.docx.

## Data Availability

The authors declare that the data supporting the findings of this study are provided in the article and its supplementary materials.
