# Supplementary material for: Potassium‐Doped Borophane Nanosheets: A Multifunctional Platform for Reversible Hydrogen Storage and Metal‐Free Hydrogen Transfer
Source: Small. 2026 Feb 23;22(22):e11090. doi: 10.1002/smll.202511090 (PMC13089104; doi:10.1002/smll.202511090)
Supplement: Supplementary file 1 — Supporting File: smll72884‐sup‐0001‐SuppMat.docx. [file SMLL-22-e11090-s001.docx]

Supplementary Materials for

**Potassium-Doped Borophane Nanosheets: A Multifunctional Platform for Reversible Hydrogen Storage and Metal-Free Hydrogen Transfer.**

Rajamohanan Sobhana Anju^1^, Pankaj Kumar^2^, Dhanaji R. Naikwadi^3^, Bettina Baumgartner^4^, Savi Chaudhary^5^, Atul Bansode^3^, Merel C. Konings^6^, F. Ariese^6^, Erdni D. Batyrev^7^, Prasad Gonugunta^8^, Vimal Chandra Srivastava^2^, Ramaswamy Murugavel^5^, N. Raveendran Shiju^1*^

*[1*] R. S. Anju, N. R. Shiju, Catalysis Engineering Group, Van ‘t Hoff Institute for Molecular Sciences, University of Amsterdam, Science Park 904, 1090GD Amsterdam, The Netherlands, E-mail:* [*n.r.shiju@uva.nl*](mailto:n.r.shiju@uva.nl)*.*

*[2] P.Kumar, V. C. Srivastava, Department of Chemical Engineering, Indian Institute of Technology Roorkee; Roorkee, India.*

*[3] D. R. Naikwadi, A. Bansode, Department of Chemical Engineering, Delft University of Technology, Van der Massweg 9, 2629 Hz Delft, The Netherlands.*

*[4] B. Baumgartner, Homogeneous, Supramolecular and Bio-Inspired Catalysis group, Van't Hoff Institute for Molecular Sciences, University of Amsterdam; Amsterdam, The Netherlands.*

*[5] S. Chaudhary, R. Murugavel, Department of Chemistry, Indian Institute of Technology Bombay, Powai, Mumbai 400076, India.*

*[6] M. C. Konings, F. Ariese, LaserLaB, Department of Physics and Astronomy, Vrije Universiteit Amsterdam; Amsterdam, The Netherlands.*

*[7] E. D. Batyrev, Tata Steel Research & Development; IJmuiden, The Netherlands.*

*[8] P. Gonugunta, Department of Material Science and Engineering, Delft University of Technology, Mekelweg 2, 2628 CD Delft, The Netherlands.*

**The file includes:**

1. **Materials and Methods**
2. **Characterization of Borophanes**
3. **Computational Calculations**
4. **Materials and Methods**

Sodium borohydride (≥ 98%), Potassium formate (99%), levulinic acid, ethyl levulinate, butyl levulinate and pyruvic acid were purchased from Merck and were used as received without further purification.

The XRD measurements were performed at TU Delft to determine the atomic arrangement in crystalline or amorphous materials by analyzing the diffraction pattern of X-rays. A Bruker D8 Advance-ECO with Bragg-Brentano geometry was used, with CuKα_1_ and Kα_2_ radiation as the X-ray source.

The XPS measurements were performed at Tata Steel IJmuiden, The Netherlands. Al-monochromated X-ray source was used at 15 mA and 15 kV to generate the X-ray photons and the emitted photoelectrons were collected from an area of 0.7 x 0.3 mm^2^. The acquired XPS spectra were processed in CASA XPS software to determine the surface composition.

XPS survey spectra of the samples were measured at TU Delft to estimate the elemental composition using PHI ESCA system supplied by the Physical Electronics, Inc., which is equipped with a non-monochromatized Aluminium (Al) Kα X-ray source
(hν = 1486.7 eV), operated at 200 W power with 13.5 kV accelerating voltage.

TEM images were recorded at TU Delft on a TEM JEOL11400 plus machine. Images were captured using a 4K camera after fine-tuning the optics.

HR-TEM images were recorded on a Thermo Scientific Themis 300 G3 microscope operating at an accelerating voltage of 300 kV. The samples were prepared by dispersing the material in isopropanol, followed by sonication and deposition onto carbon-coated copper grids, which were then air-dried.

TGA analyses was performed at Delft University, from 30 to 860 °C, heating rate of 10 °C⋅min-1 using a Perkin Elmer TGA 4000. SEM-EDS were recorded using JEOL (JSM-IT800)

Transmission infrared (IR) spectra were recorded at University of Amsterdam, using an Invenio R Fourier-transform infrared (FTIR) spectrometer (Bruker Optics, Ettlingen, Germany) equipped with a DLa-TGS detector. Spectra were collected in the range of 4000-600 cm^-1^ with a resolution of 4 cm^-1^. For all measurements, KBr pellets were prepared with a total mass of 100 mg, containing 1 wt% of the sample.

In situ ATR FTIR spectra were recorded using Fourier transform-infrared (FTIR) spectroscopy (Bruker InvenioR FTIR spectrometer with a N2-cooled MCT detector) in attenuated total reflection (ATR) mode. Si ATR crystals (20 x 10 x 0.5 mm, 45°, typical penetration depth at 1600 cm^-1^ around 520 nm, and effective pathlength around 9 µm) were cut from double side polished Si wafer (Siegert). (same references as before) LA solutions (add concentration) were applied with 1 mL min-1 flow using a peristaltic pump (Masterflex) and 1/16’’ PFA tubing. Prior to analyte application, the flow cell was flushed with pure solvent (ethanol or toluene) until a stable baseline was achieved. Si ATR crystals were coated with BH from methanol suspensions and dried at 80 °C for 30 min before being placed in the home-built aluminum flow cell in the FTIR spectrometer. The interferometer and sample compartment of the FTIR spectrometer was flushed with N_2_. For each spectrum, 32 scans were averaged. FTIR spectra were derived from single channel spectra and further processed in MATLAB. For each experiment a freshly prepared BH-coated ATR crystal was used.

Raman measurements were performed at Vrije Universiteit Amsterdam with an InVia Reflex Renishaw system. A frequency doubled Nd:YAG laser of 532 nm with a grating of 1200 l/mm was used (range between 104 and 2735 cm^-1^). The system is operated with Renishaw WIRE^TM^ software. Particles of interest were measured with a 50x objective, with an exposure time of 1 s, accumulations between 10 and 20 and varying laser power of between 5 and 10 % (depending on the intensity of the signal).

The spectra were slightly smoothed, background-corrected, and normalized using MATLAB (version R2024a, 24.1.0.2603908). Smoothing was performed using the 'Smooth Data' task with a Savitzky-Golay polynomial filter, applying a moving window with a center value of 5. Background correction was achieved by subtracting a baseline estimated using the 'backcor' function. A second-order polynomial with an asymmetric truncated quadratic cost function and a threshold of 0.1 was used for all spectra, except for measurements on carbon black, which required a third-order polynomial due to more intense fluorescence. In some cases, the constant term in the polynomial was adjusted to shift the polynomial up or down before subtraction to preserve peak integrity. Finally, the spectra were normalized by rescaling them to a 0–100 range using MATLAB’s 'rescale' function.

For ICP-OES analysis, the BH sheets were fully digested in aqua regia to ensure complete dissolution, and the resulting solutions were analyzed using a SPECTRO ARCOS EOP instrument equipped with a modified Lichte nebulizer and a mini-cyclone spray chamber.

**Synthesis of Borophanes (BHs) and Borophene Oxides (BOs)**

**(i) Synthesis of BH:** NaBH_4_ and HCOOK were thoroughly mixed in molar ratios of 9:1, 6:1, 3:1, or 2:1 using a mortar and pestle. The resulting mixture was transferred to a quartz boat and placed in a tubular furnace. The mixture was then heated to 600 °C under a nitrogen flow (50 mL min⁻¹), following the heating profile shown in Scheme S1a. After cooling to room temperature, the material was carefully exposed to air and left under ambient conditions for 3-4 h. During this period, alkali metals or metal clusters formed during the reaction developed a tarnished layer due to surface oxidation or hydroxide formation. The material was then finely ground and gradually treated with 30 mL of water under sonication (10 min) to avoid vigorous reactions. The suspension was filtered, washed thoroughly with cold and hot water to remove oxalates and any carbonates; and dried in an oven at 120 °C for 3 h. The proposed chemical reactions are presented in equations (1-4) and Scheme S1.

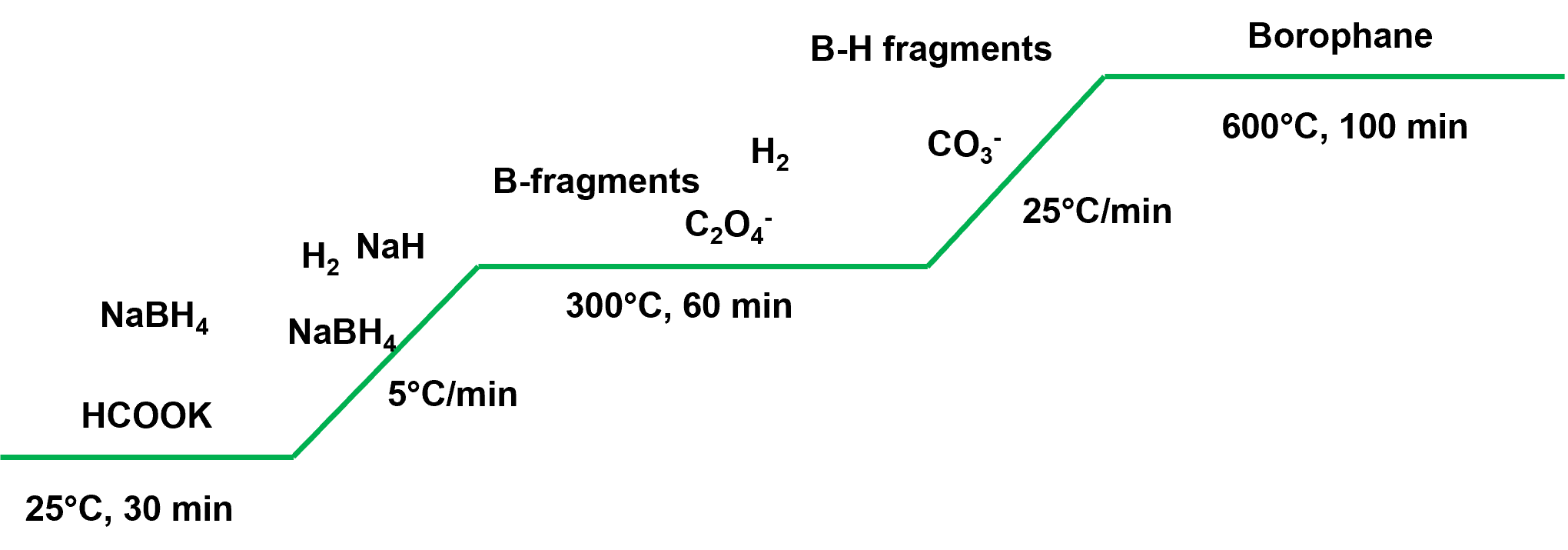


Scheme S1: Schematic illustration of the formation of BHs (Molar ratio between NaBH_4_ and HCOOK is 2:1or 3:1 or 6:1 or 9:1).

**(ii) Reduction reactions**

Levulinic acid (500 mg) was dissolved in toluene and placed in a round-bottom flask. BH(6:1) (400 mg) was added, and the reaction mixture was heated to 80 °C and stirred for 14 h. Upon completion, the mixture was filtered to remove the spent BH(6:1), and the filtrate was washed thoroughly. The product, γ-valerolactone (GVL), and characterized by ¹H and ¹³C NMR spectroscopy.

**(iii) High-pressure regeneration experiments**

The reactions were carried out by loading the spent BH(6:1) into an autoclave, which was then degassed and pressurized with hydrogen gas (50-80 bar). The autoclave was placed in a Parr 5000 multi-reaction system and heated to 200 °C for the required reaction time.

**Note**: Under identical reaction conditions, when nitrogen gas was used in place of hydrogen; subsequent testing showed no observable LA reduction.

The experiments were conducted by varying the H_2_ pressure from atmospheric pressure up to 80 bar and the reaction temperature from 80 to 200 °C. However, no measurable hydrogenation was observed at pressures below 50 bar or at temperatures below 200 °C.

**(iv) Analysis of released gas from BH(6:1)**

100 mg of BH(6:1) was placed in a quartz reactor and heated in presence of N_2_ from 30- 300 ℃ and analyzed the released gas via GC-TCD. It was found that only hydrogen gas is formed during the heating process.

**(v) Calculation for maximum gravimetric hydrogen storage capacity (as wt% H_2_)**

For B_53_H_37_O_10_KC_7_, the maximum gravimetric capacity as wt% H_2_ (i.e., assuming all 37 H atoms are released as H_2_) is 839.39 g/mol (Molar mass).

Mass of hydrogen present = 37 * 1.008 = 37.296 g/mol

Wt % H_2_ = (37.296/ 893.39) * 100 = 4.175 %

1. **Characterization of Borophanes (BHs)**

**
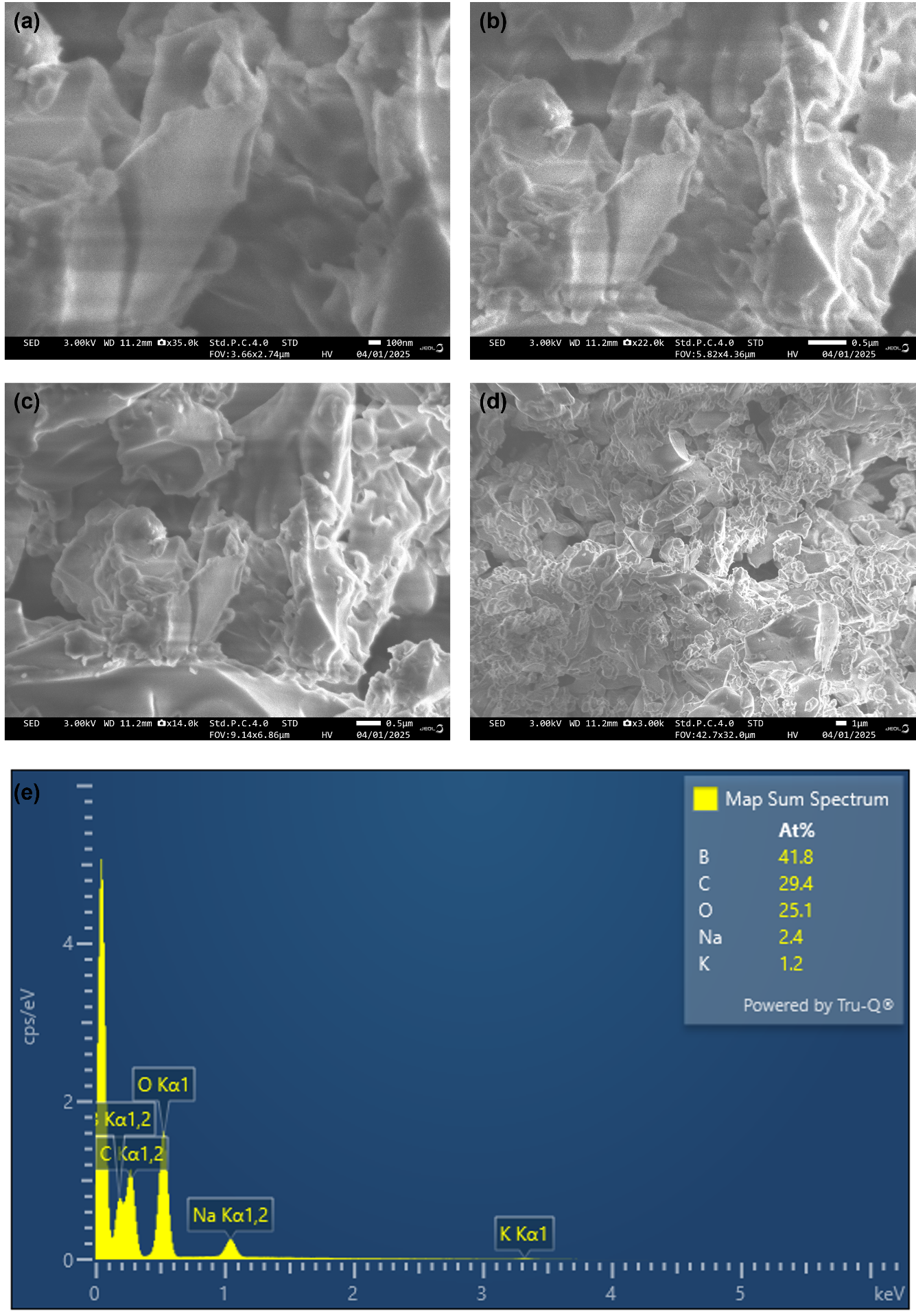
**

**Figure S1:** (a-d) SEM images of BH(6:1); (e) Quantitative elemental composition (atomic %) obtained from the SEM-EDS mapping of BH(6:1).


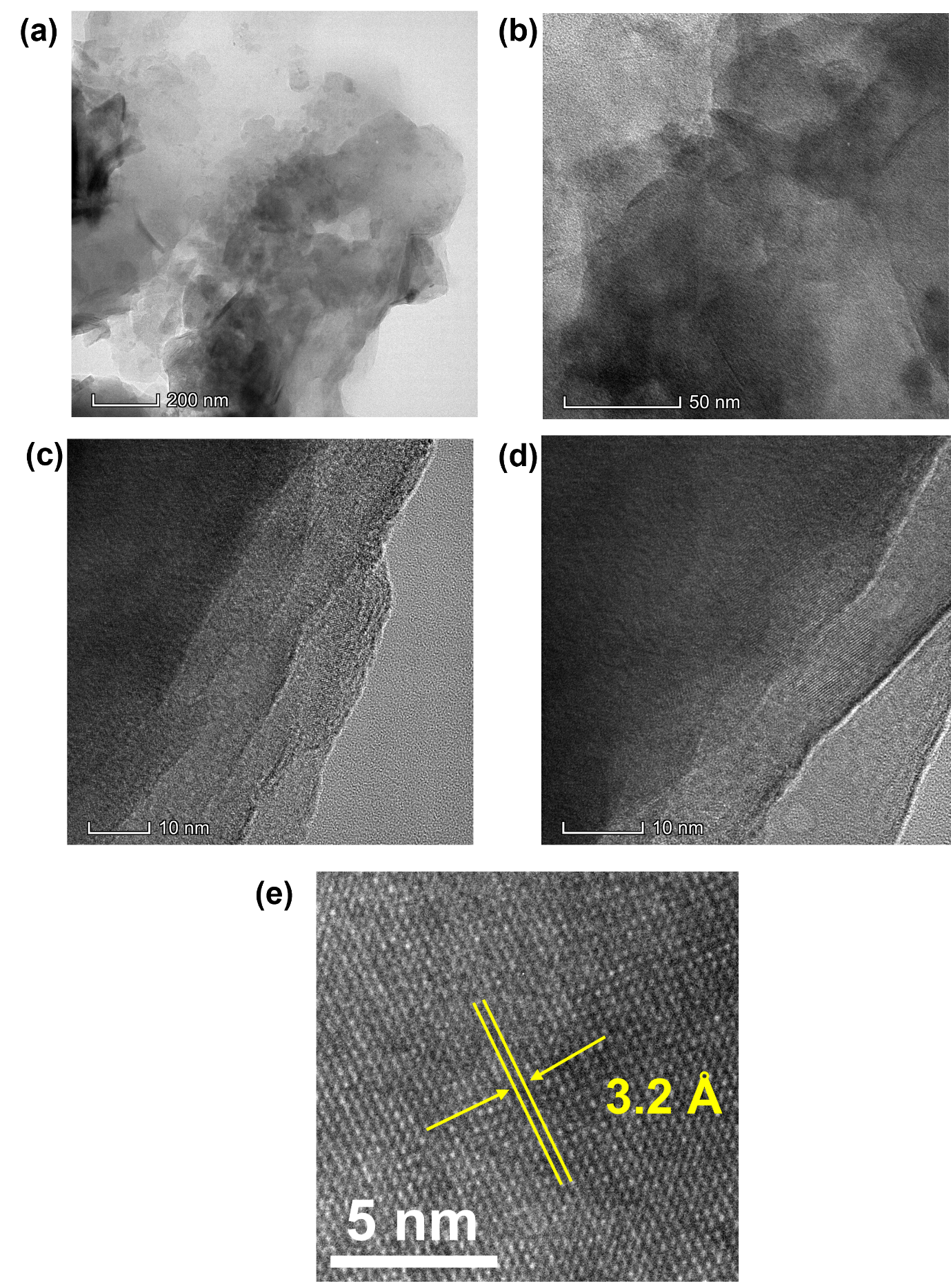


**Figure S2:** (a-d) HR-TEM images of BH(6:1); (e) locally magnified view of the HR-TEM image given in figure 1d.

**
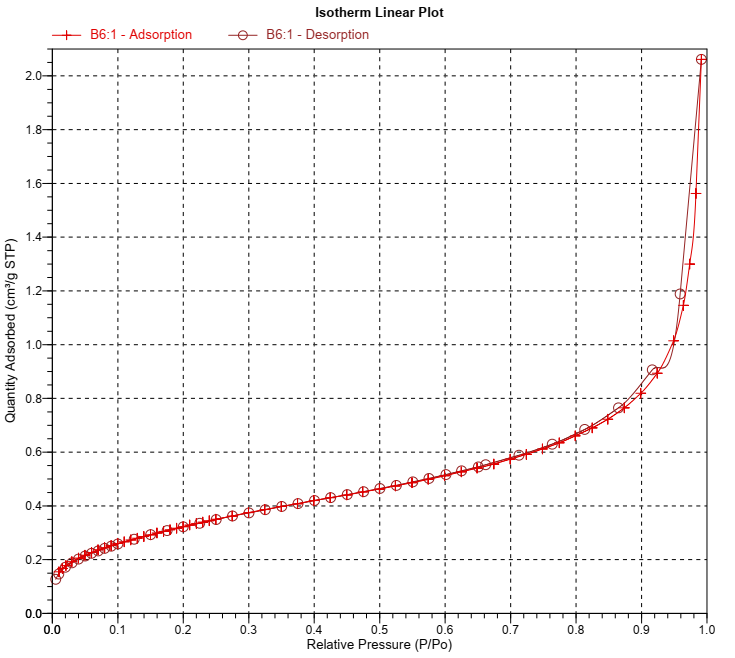
**

**Figure S3:** BET analysis of BH(6:1)


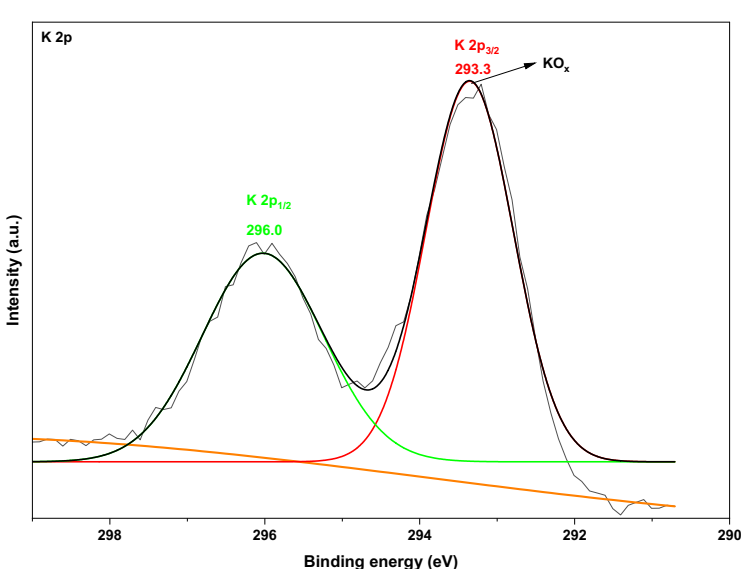


**Figure S4**: K 2p XPS spectra of BH(6:1).


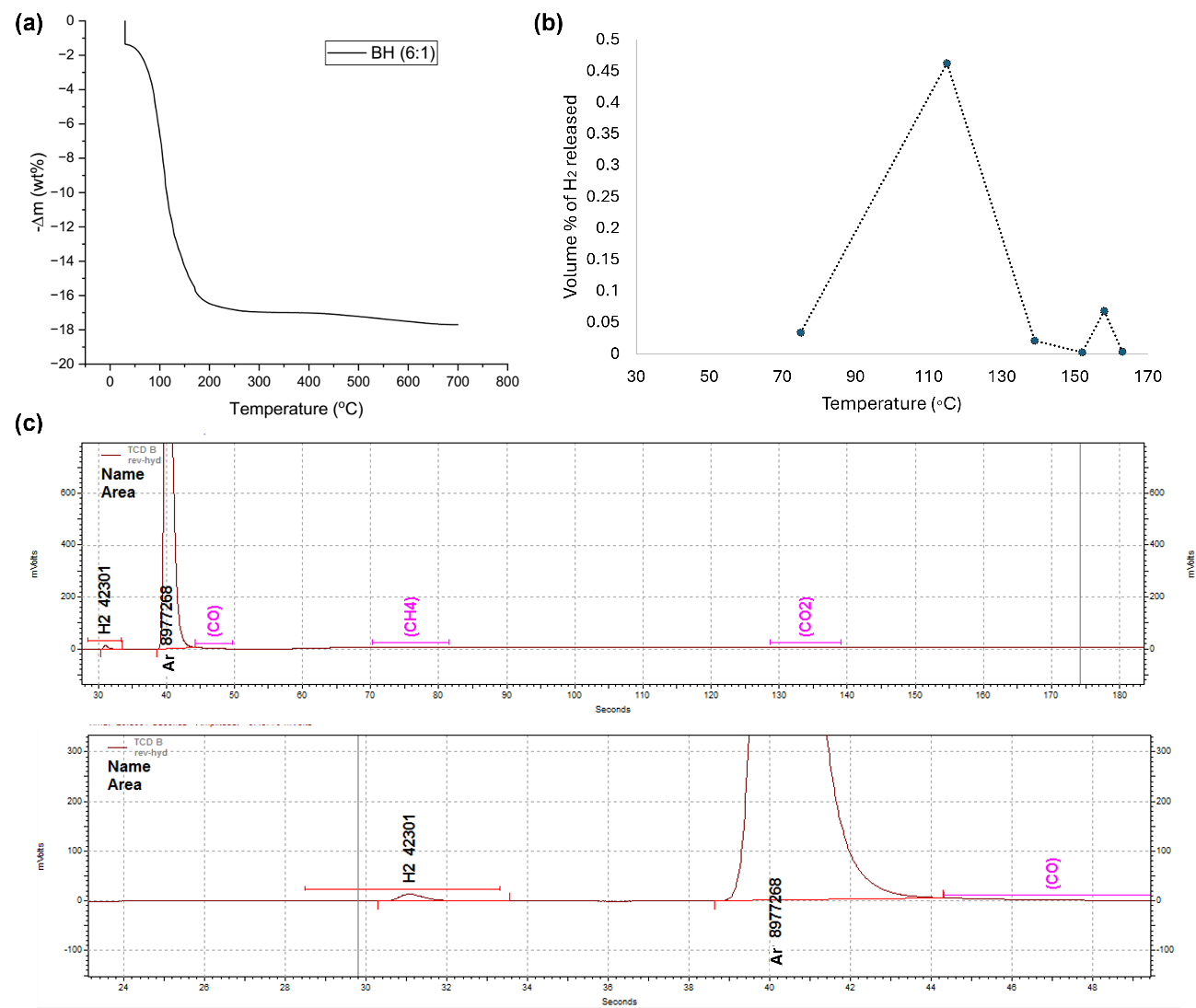


**Figure S5**: (a) TGA analysis curve of BH(6:1); (b) Plot of temperature‐programmed H_2_ evolution from BH(6:1). A 100 mg sample of BH(6:1) was loaded in a quartz reactor and heated from 30 to 300 °C (heating rate was not constant), while the outlet gas composition was monitored by online GC-TCD. The curve shows the H_2_ concentration in the effluent (vol%) as a function of temperature; (c) Representative GC-TCD chromatogram of temperature‐programmed H_2_ evolution from BH(6:1), showing a single peak corresponding to H_2_ and no additional gaseous products detectable within the sensitivity of the method.


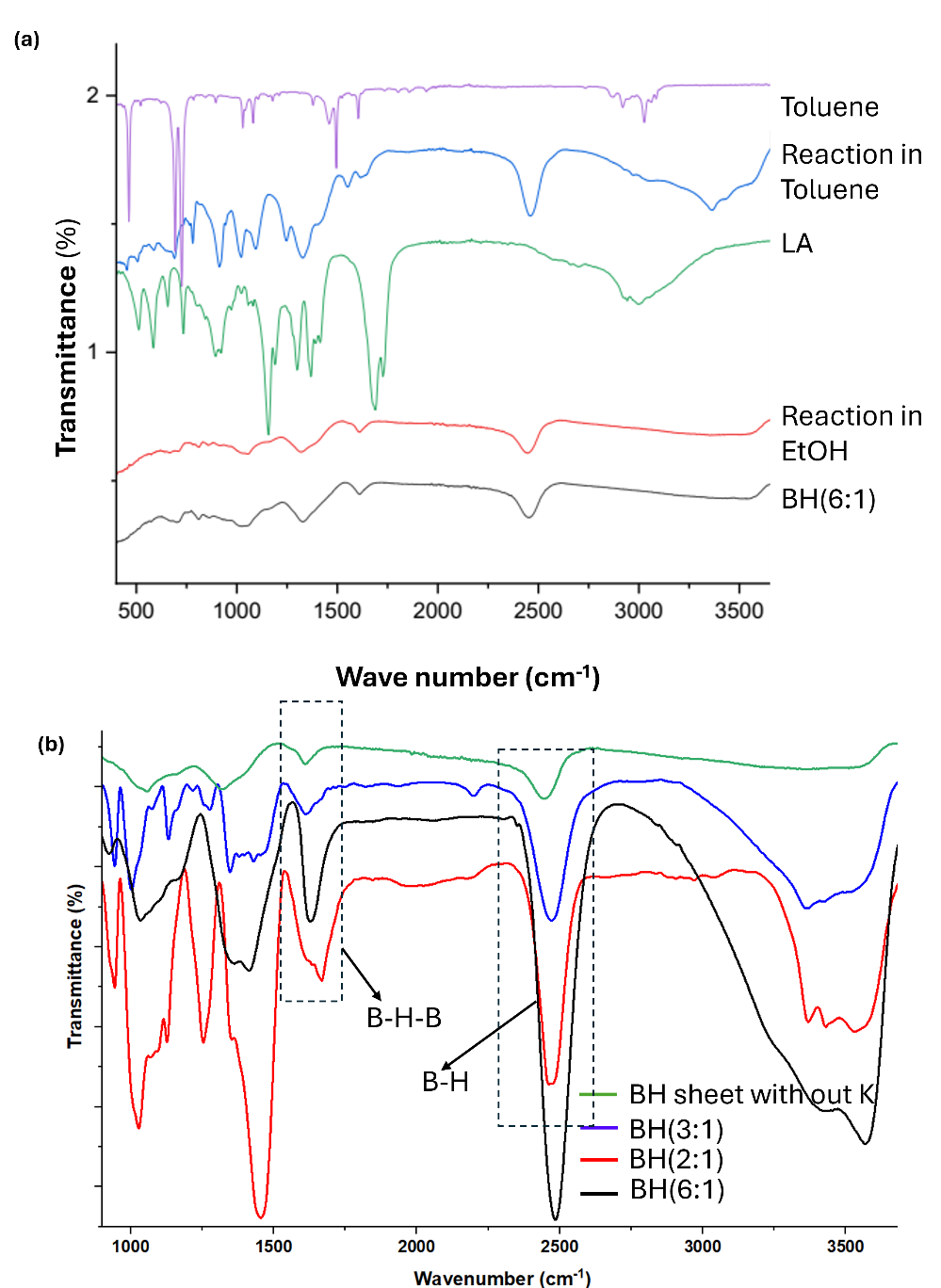


**Figure S6**: (a) Comparison of Ex-situ FTIR spectra of BH(6:1), toluene, LA, and spectra of BH(6:1) recorded after performing the reaction with LA in toluene and ethanol followed by filtration and air-drying; (b) Comparison of FTIR spectra of BH(6:1), BH(3:1), BH(2:1), and the potassium-free BH sheet synthesized by heating NaBH_4_ under a H_2_ atmosphere. All spectra were acquired using equal sample masses to enable direct quantitative comparison.


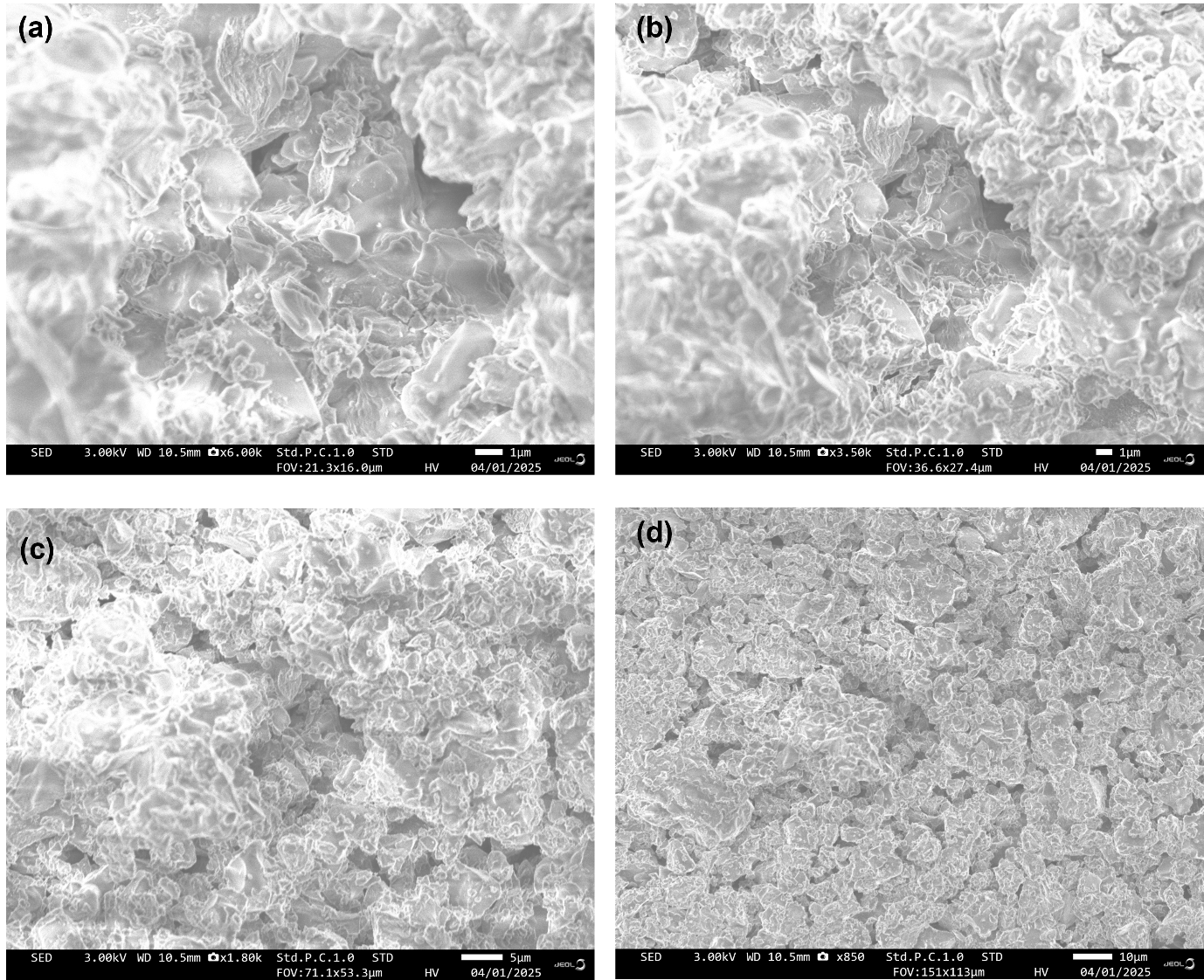


**Figure S7**: (a-d) SEM images of spent BH(6:1)

**
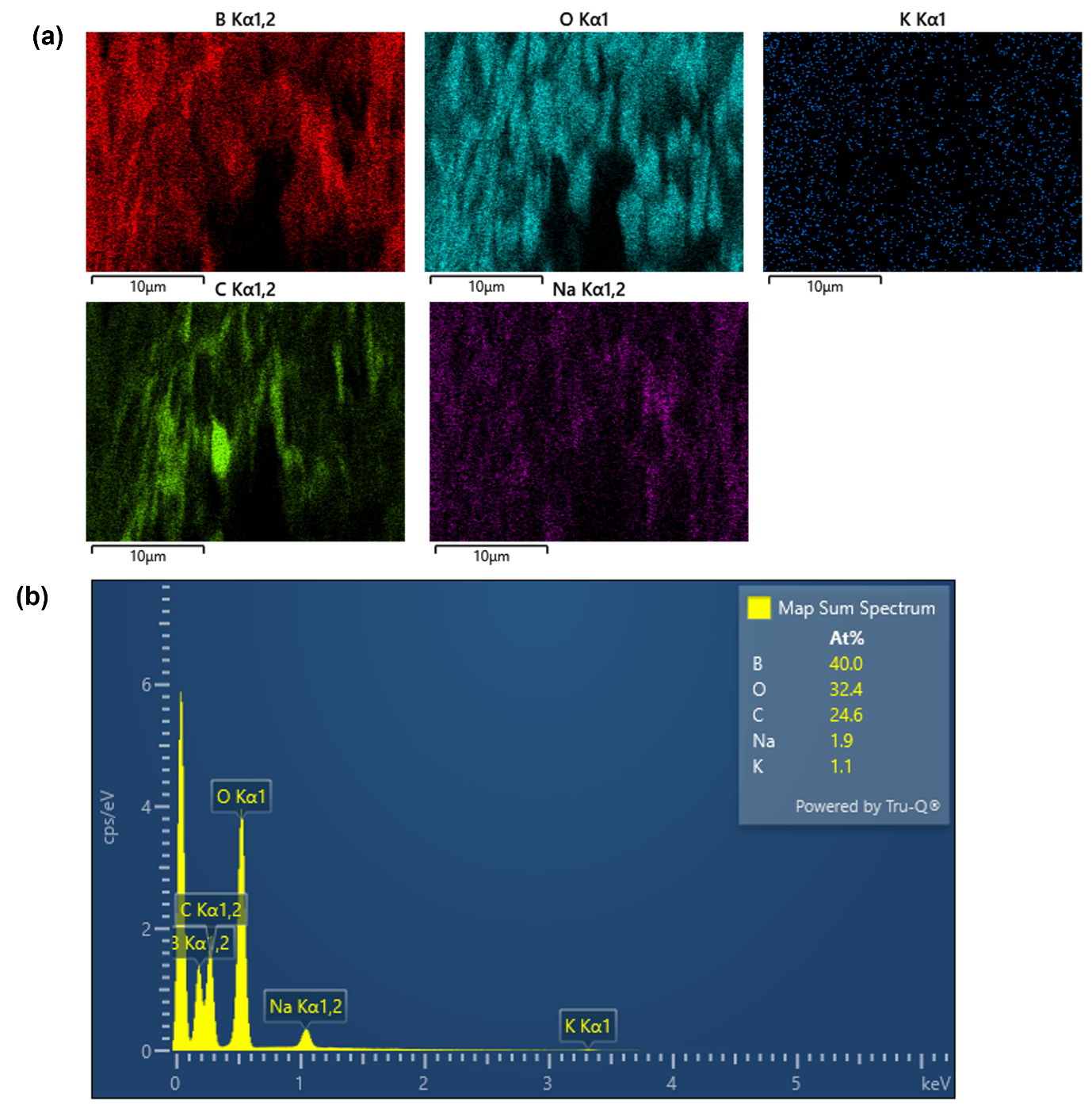
**

**Figure S8**: (a-b) SEM-EDS mapping and quantitative elemental composition (atomic %) obtained from the mapped region of spent BH(6:1).


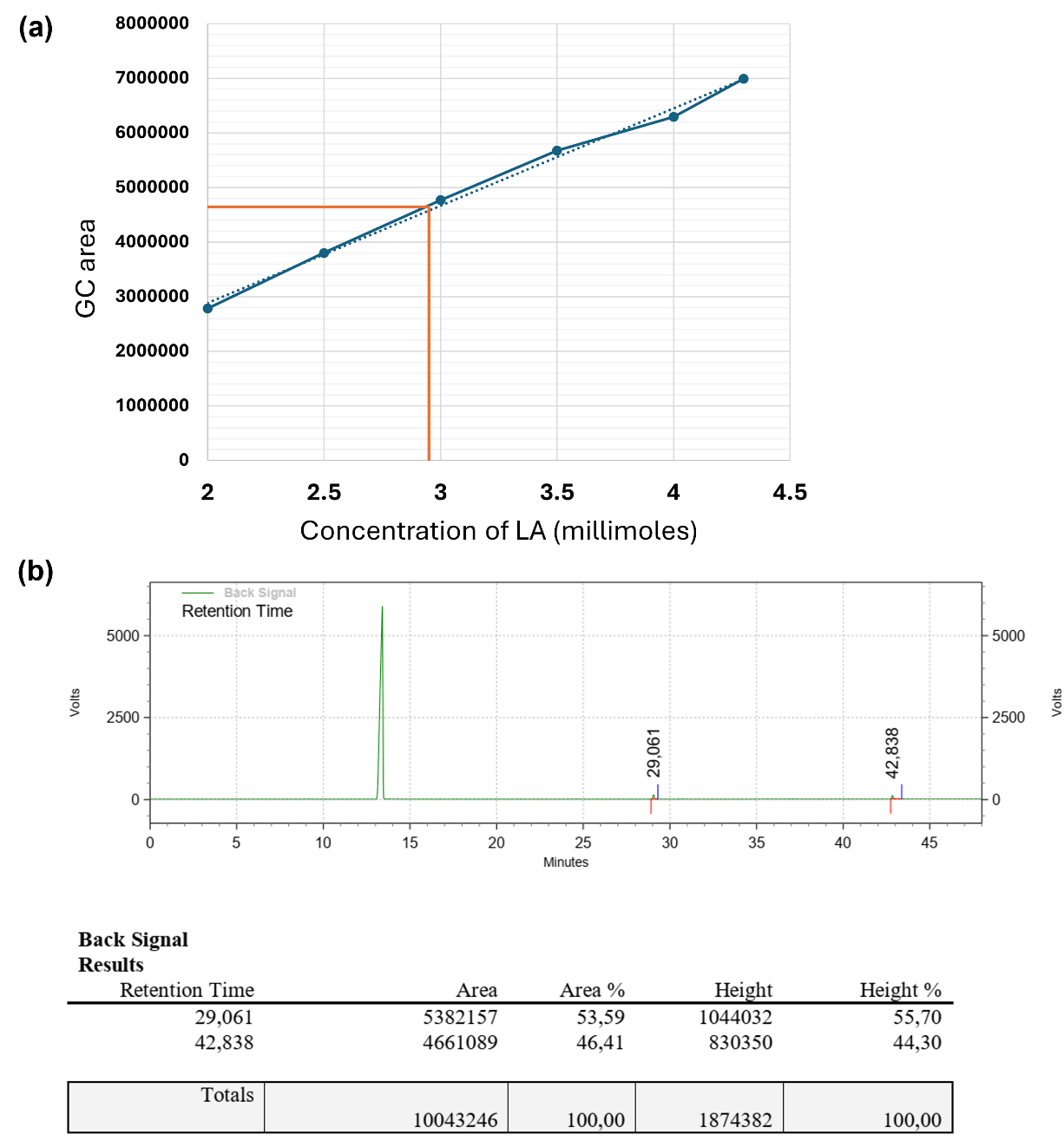


**Figure S9**: (a) Calibration of LA performed by recording GC peak areas for a series of standard solutions spanning 2.0-4.3 mmol LA. The LA concentration in the reaction samples was then determined by interpolating the measured peak areas on the resulting calibration curve; (b) GC-FID chromatogram of the reaction mixture containing both unreacted LA (2.8 mmol) and GVL obtained after 14 h of reaction between spent BH(6:1) and 4.3 mmol of LA.

**
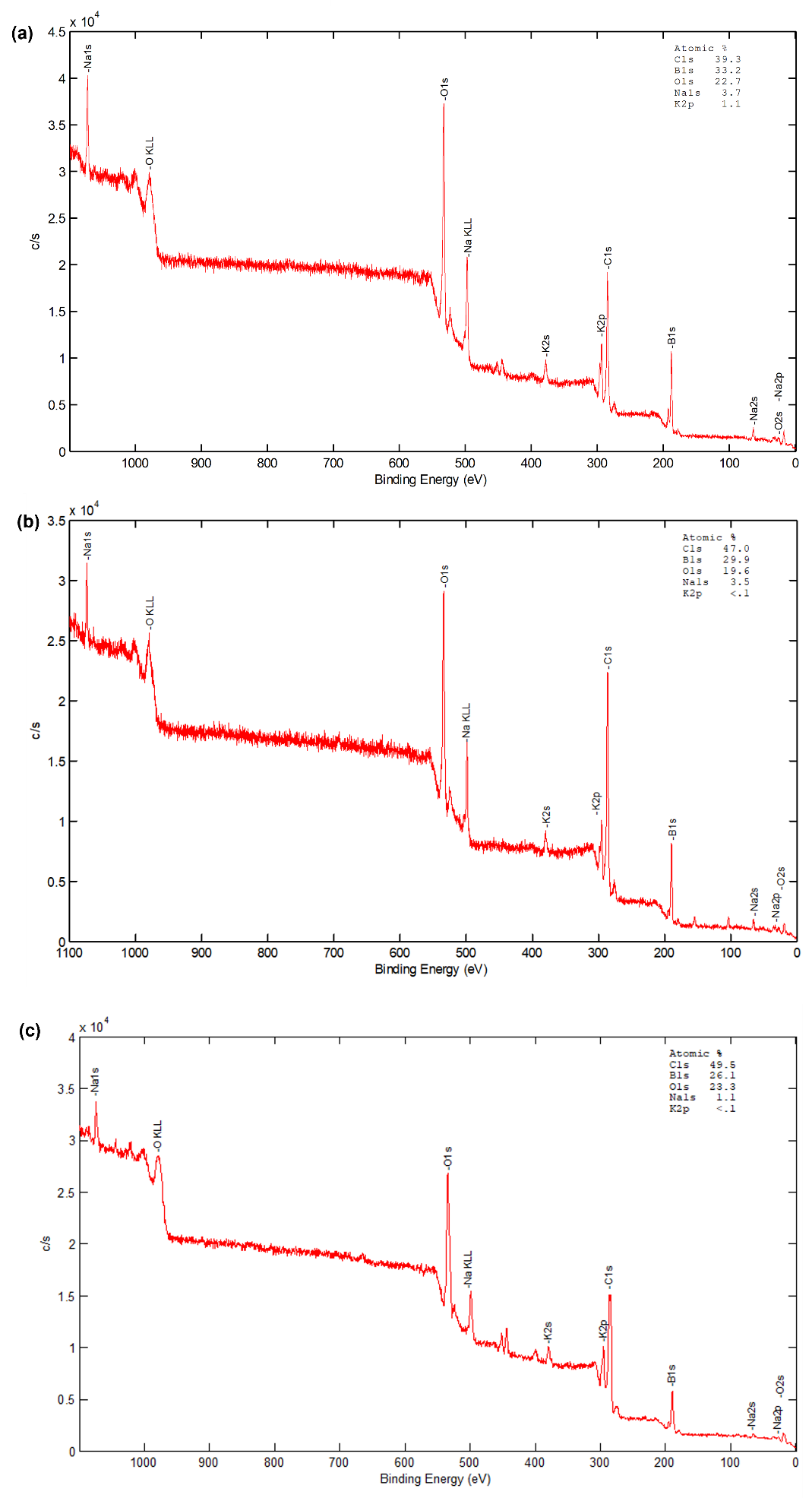
**

**Figure S10**: XPS-derived surface atomic compositions of (a) BH(6:1), (b) spent BH(6:1), and (c) regenerated BH(6:1), showing a decrease in boron content from 33 at% in BH(6:1) to 26 at% in regenerated BH(6:1).

**
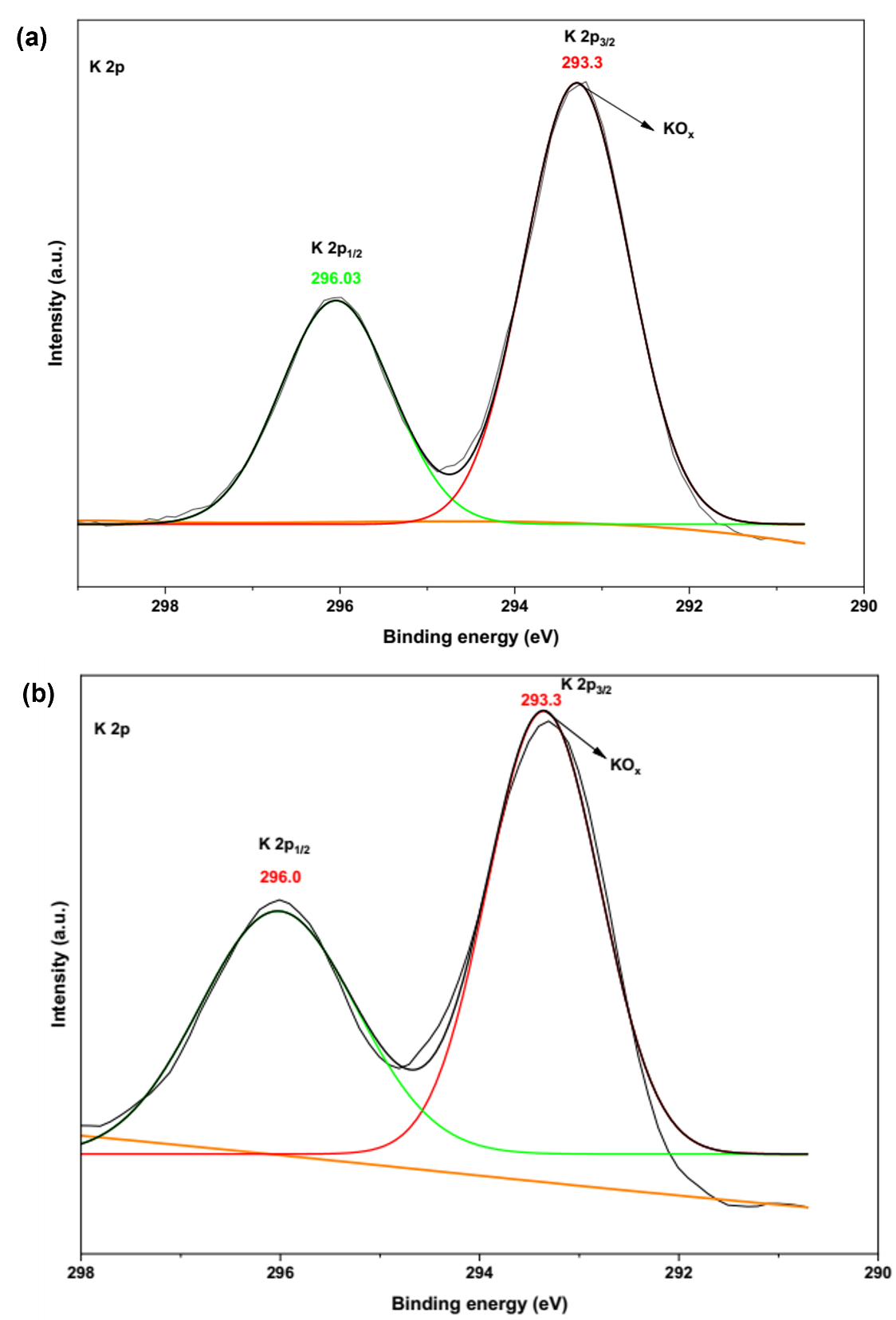
**

**Figure S11**: K 2p XPS of (a) spent BH(6:1) and (b) regenerated BH(6:1)


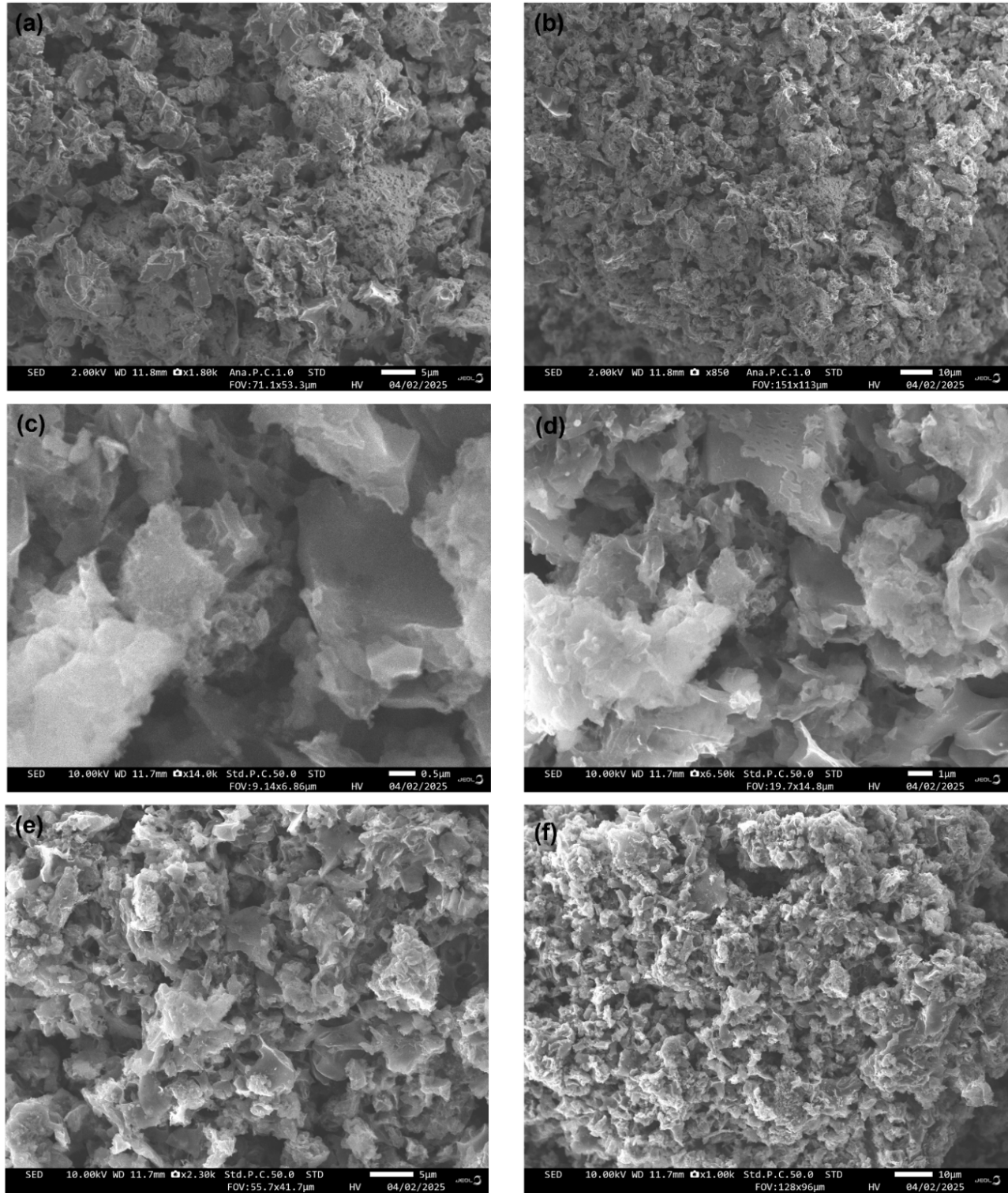


**Figure S12**: SEM analysis of regenerated BH(6:1)

**
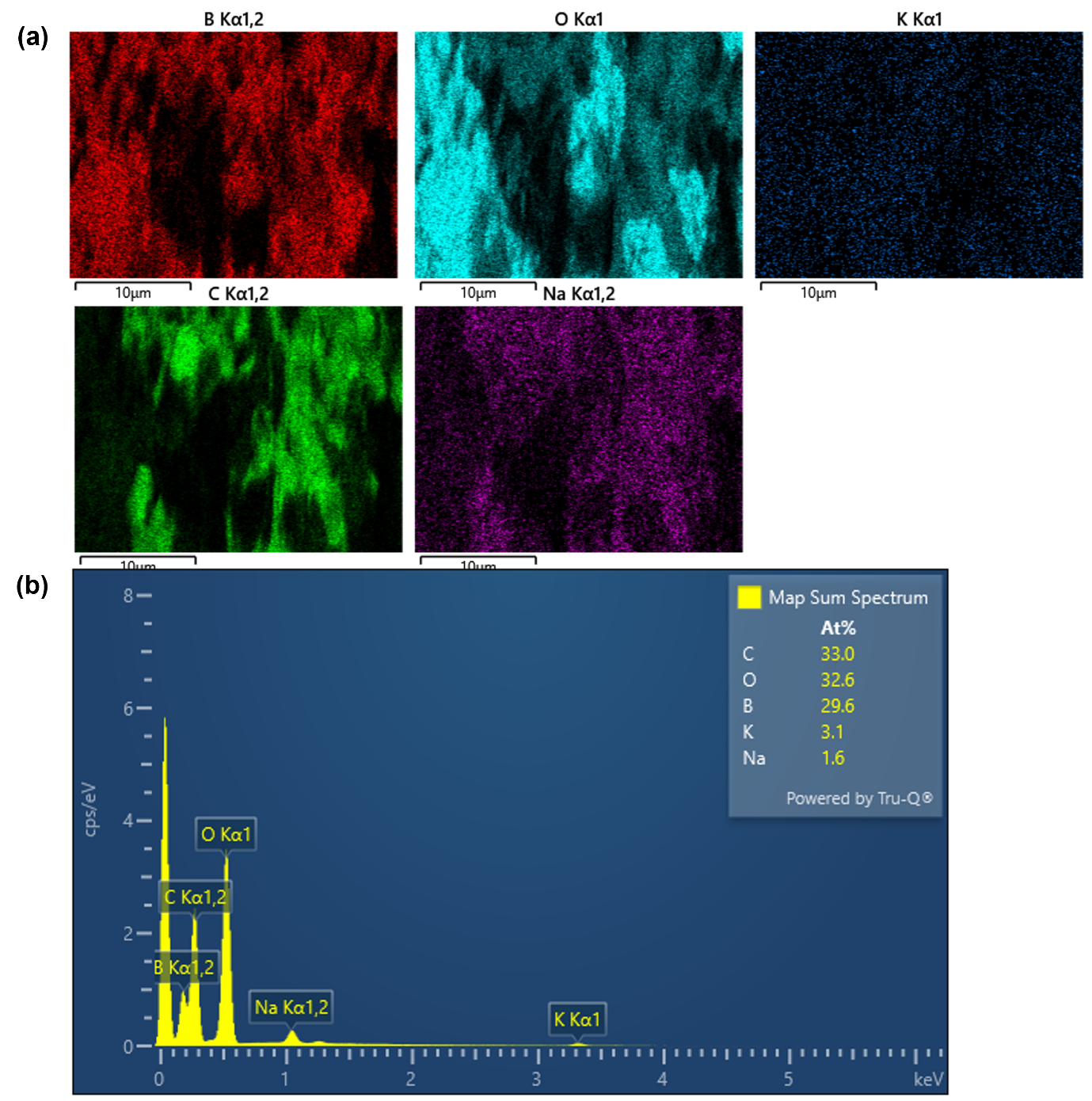
**

**Figure S13**: EDS analysis of regenerated BH(6:1)


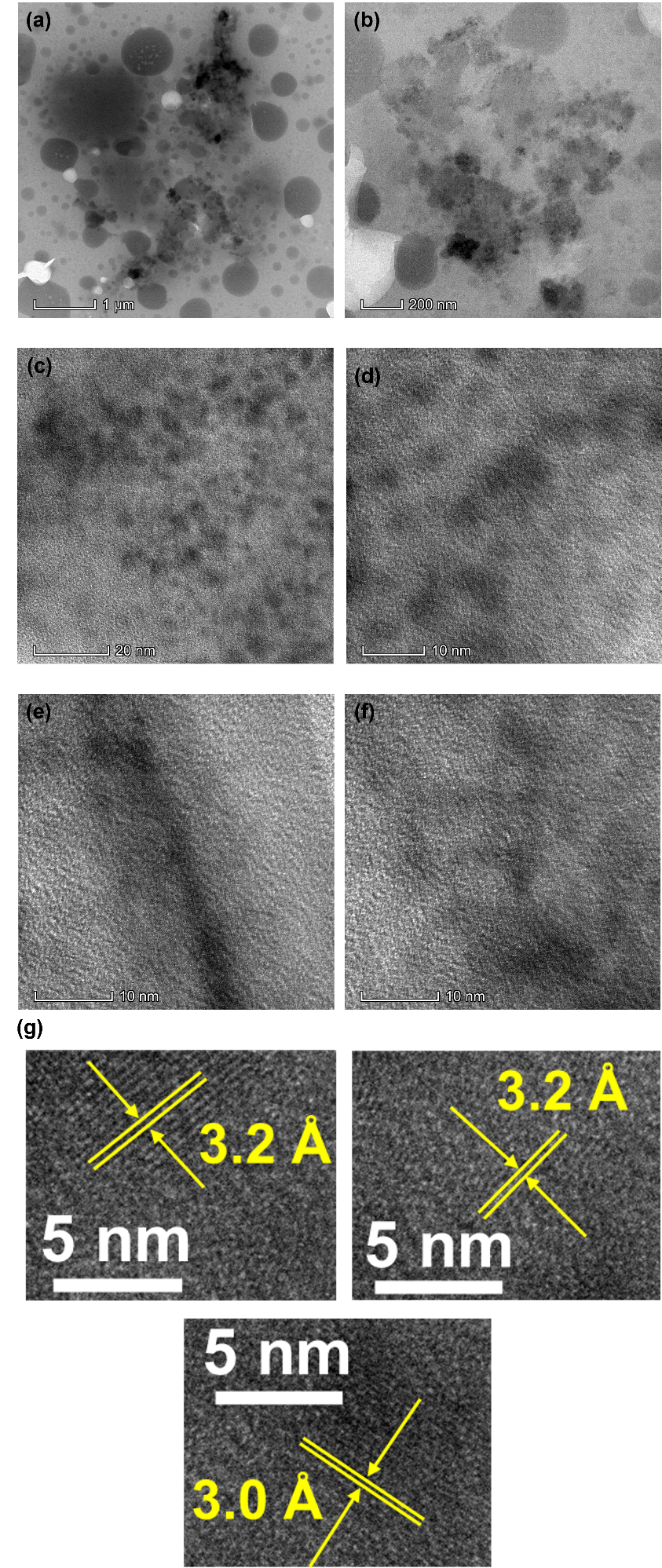


**Figure S14**: (a-f) HR-TEM analysis of spent BH(6:1); (g) locally magnified images of HR-TEM image shown in figure 4j.

**
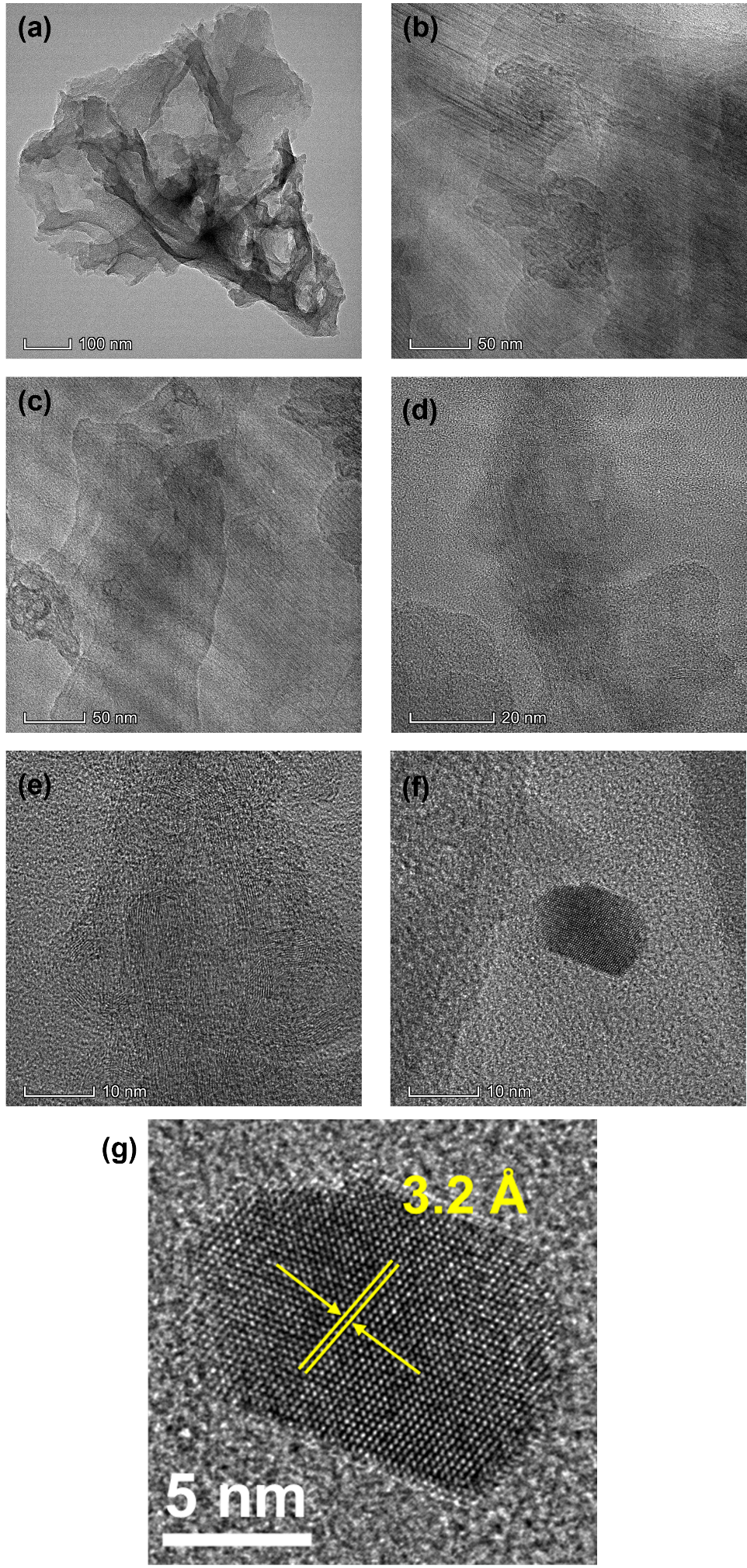
**

**Figure S15**: (a-f) HR-TEM analysis of regenerated BH(6:1) and locally magnified image of HR-TEM image given in figure 4k.

**
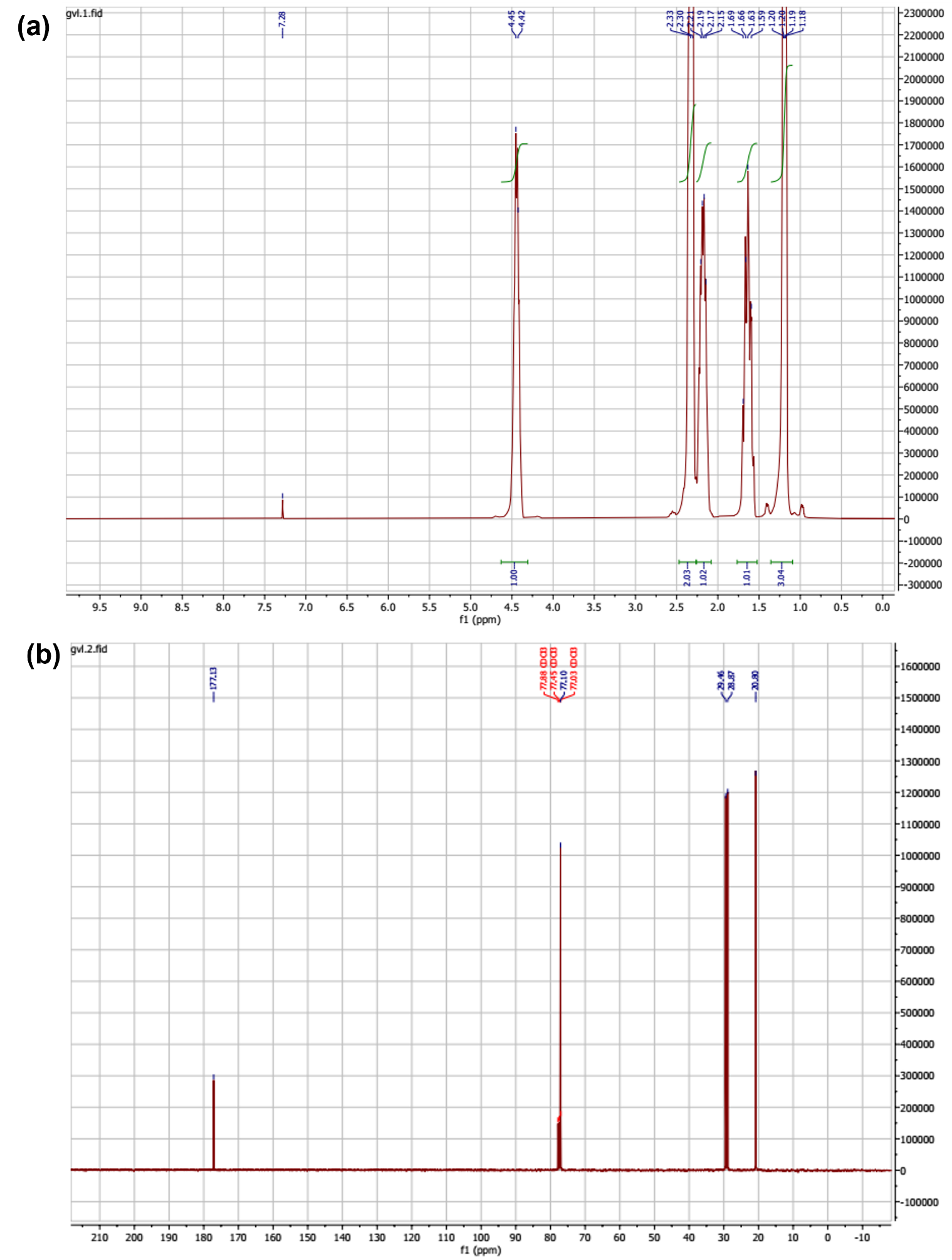
**

**Figure S16**: (a) ^1^H and (b) ^13^C NMR of GVL

**Table S1**: Reduction of ethyl levulinate, butyl levulinate and pyruvic acid using BH(6:1).

| Entry | Reagent | Solvent | Product | Yield |
| --- | --- | --- | --- | --- |
| 1 | Ethyl levulinate | Toluene | -- | -- |
| 2 | Ethyl levulinate | Ethanol | -- | -- |
| 3 | Ethyl levulinate | Toluene-Ethanol mixture (1:1) | -- | -- |
| 4 | Butyl levulinate | Toluene | -- | -- |
| 5 | Pyruvic acid | Toluene | Lactic acid | 67% |
| Reaction conditions: T = 80 °C; time = 14 h; yield. | | | | |

**Table S2**: ICP-OES derived bulk elemental composition of BH(6:1), spent BH(6:1), and regenerated BH(6:1), quantifying the B, Na, and K contents in each sample.

| **Name** | **Weight (%)** | | |
| --- | --- | --- | --- |
|  | **B** | **Na** | **K** |
| **BH** | 24.8 | 4.1 | 11.6 |
| **Spent BH (6:1)** | 22.2 | 3.8 | 10.6 |
| **Regenerated BH(6:1)** | 19.5 | 2.2 | 10.2 |

**3. Computational Studies**

The experimental findings are supported with DFT calculations using a methodology analogous to the one used by Kumar et al. (2024) and Kumar et al. (2025) using Quantum Espresso 7.2 software (*1*–*3*). The electronic structures were visualized using a plane wave basis set and the Perdew-Burke-Ernzerhof (PBE) exchange correlation with Projector-Augmented-Wave (PAW) Pseudopotentials (*4*–*6*).

**Optimization of Borophane:** The properties of bulk and active surface were calculated by performing structural relaxation calculations using kinetic energy cut-off for wavefunctions (ecutwfc) and the kinetic energy cut-off for charge density and potential (ecutrho) of 70 Ry and 700 Ry, respectively. ecutwfc is the maximum number of plane waves used for defining the wavefunction of the system (catalyst and/or reacting species). ecutrho is the number of plane waves used for defining the charge density and potential of the system (catalyst and/or reacting species). The thresholds of force and energy were set to 1×10^-4^ Ry/atom and 1×10^-3^ Ry/atom, respectively. High verbosity was used to calculate total stress and forces. The bulk was relaxed using convergence criteria of 1×10^-8^ Ry with 108 atoms in a vacuum of 12 Å to prevent periodic image interaction. Convergence criteria are the representations of the values of energy or force on atoms having value less than the defined value, which gets repeated at least 2 times in consecutive 2 scf calculations. Brillouin zones (zones in 3-D space used to understand the behavior of electrons and excited states) were sampled using 4 4 1 Monkhorst-Pack k-points with Gaussian smearing (*7*).

The adsorption of LA on the active surface of the bulk catalyst was analyzed using the following energy-based indicators:

(i) Energy of adsorption (*E_adsorption_*) on the active surface of catalyst:


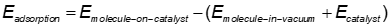
 (1)

(ii) Energy of desorption (*E_desorption_*) from the active surface of catalyst:


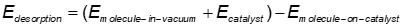
 (2)

Where
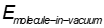
,
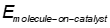
and
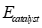
are the energies of LA molecule in bulk phase (vacuum), the total energy after adsorption of molecule, and the energy of the catalyst, respectively. The creation and visualization of the structures and the calculation of angles and bond length of the optimized structure were performed using VESTA, Burai, and Xcrysden (*8*–*10*). The free energy change for each step was separately calculated using computational hydrogen electrode model (*11*)*.* Mulliken charge analysis of the optimised structures was performed by re-performing the energy calculations on optimised structures in Gaussian 16W and Gauss View 6 software. Fukui function calculations were calculated using UCA-FUKUI V2 software (12).


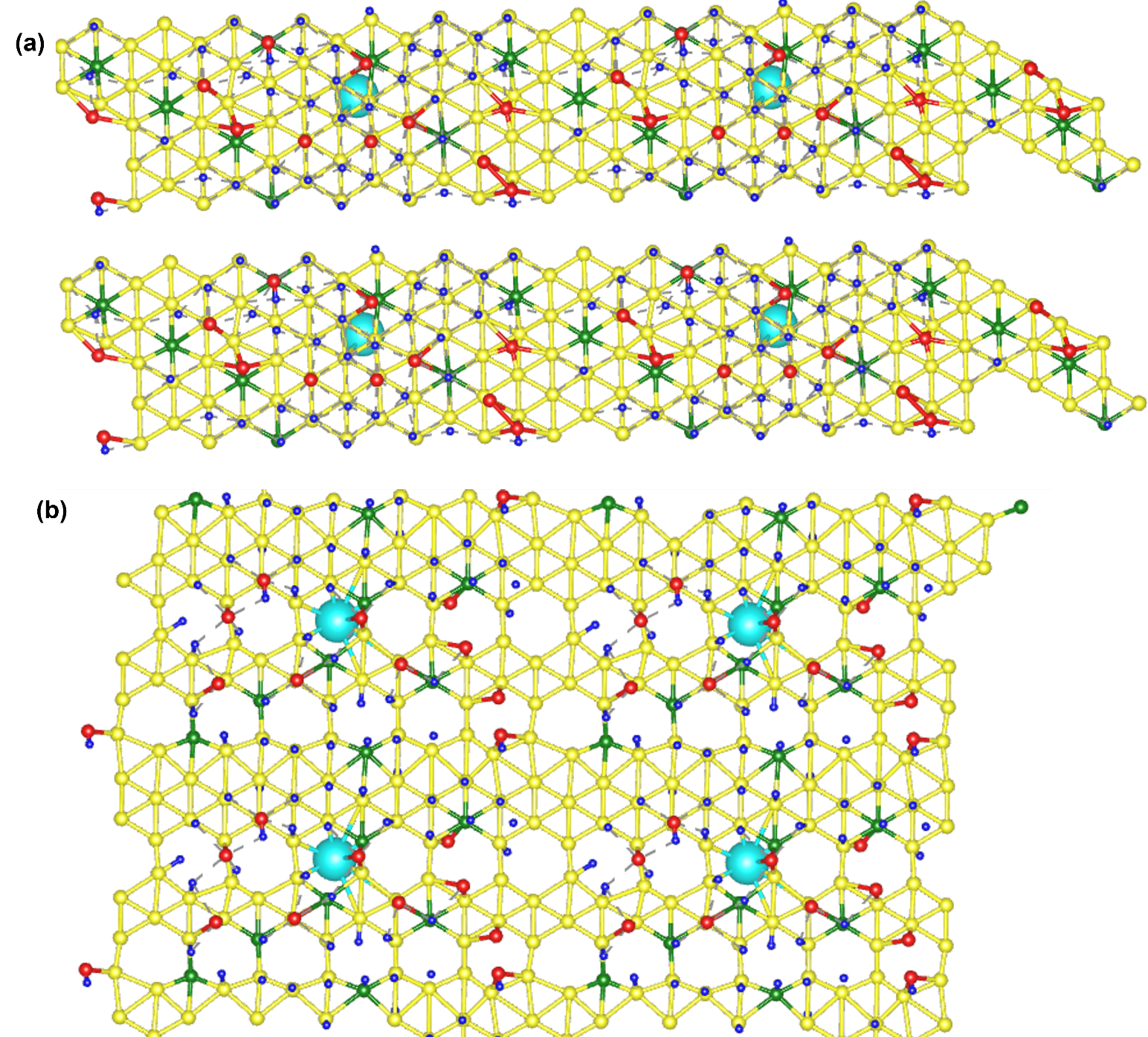


**Figure S17:** DFT optimized structure of (a) Striped BH and (b) X_3_ BH.

**
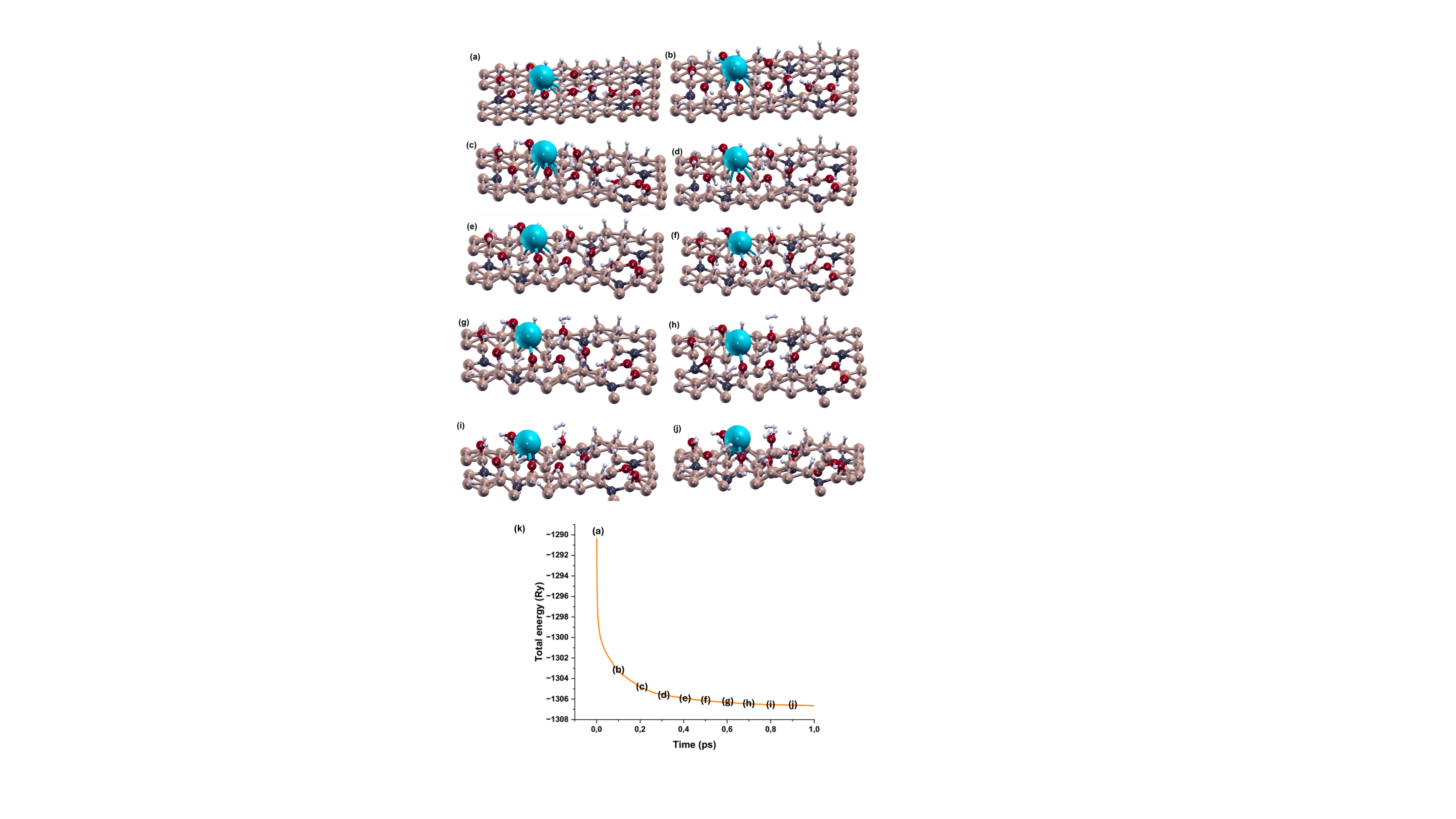
**

**Figure S18:** MD simulations of β_12_ structure by employing Anderson Thermostat at 250K on N-V-T ensemble; Fig.(a-j) shows the progression of MD simulations; (k) Stability curve for the MD calculations (energy vs time).


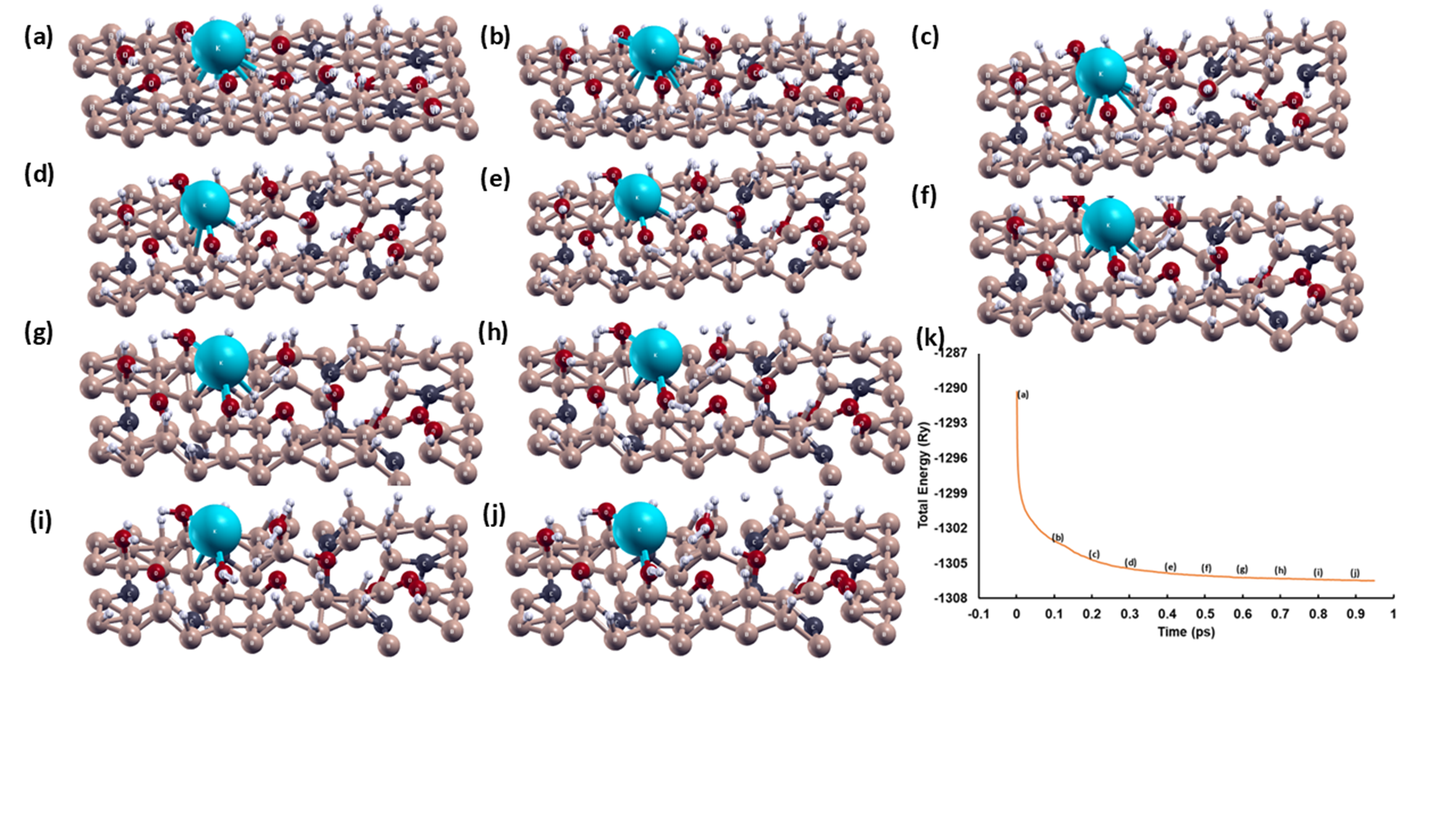


**Figure S19:** MD simulations of β_12_ structure by employing Anderson Thermostat at 300K on N-V-T ensemble; Fig.(a-j) shows the progression of MD simulations; (k) Stability curve for the MD calculations (energy vs time).


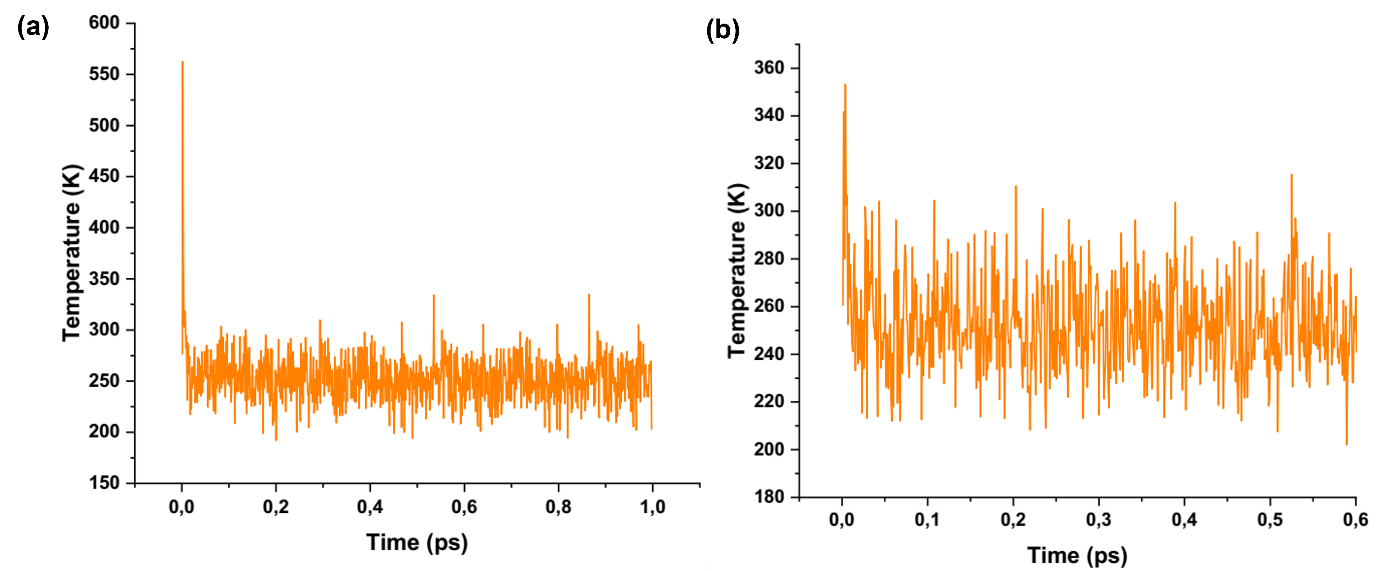

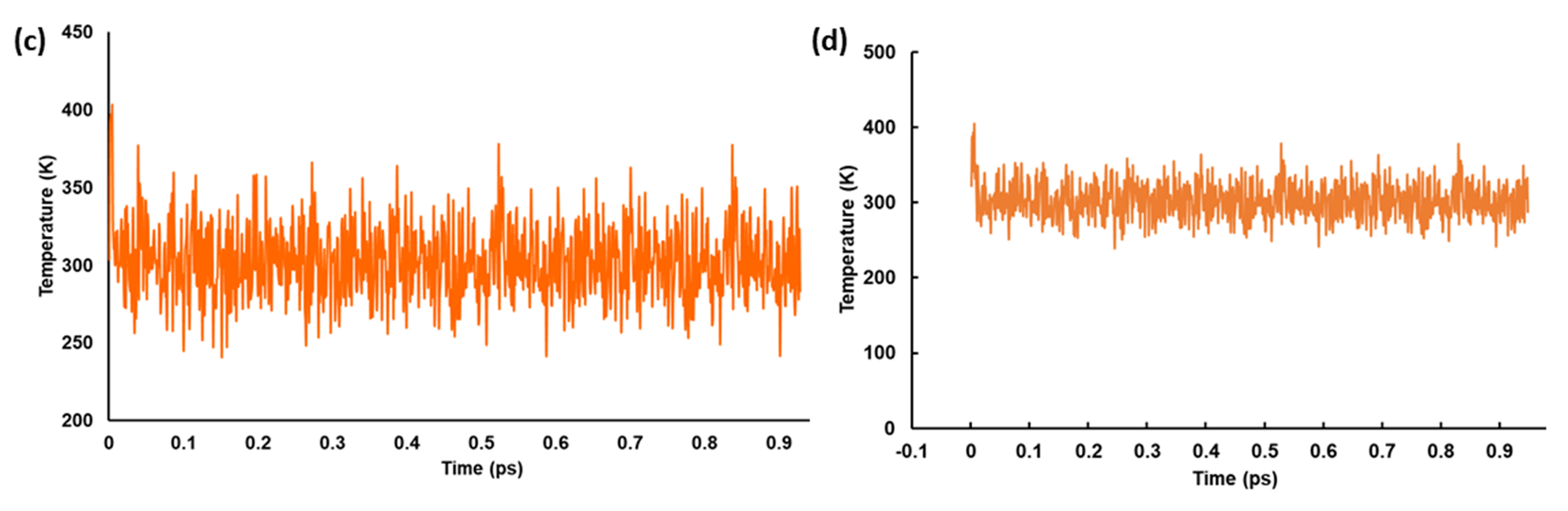
**Figure S20:** (a)Temperature vs time curve for MD simulation of BH only at 250K; (b) Temperature vs time curve for MD simulation of LA interaction with BH at 250K; (c)Temperature vs time curve for MD simulation of BH only at 300K; (d) Temperature vs time curve for MD simulation of LA interaction with BH at 300K.

**
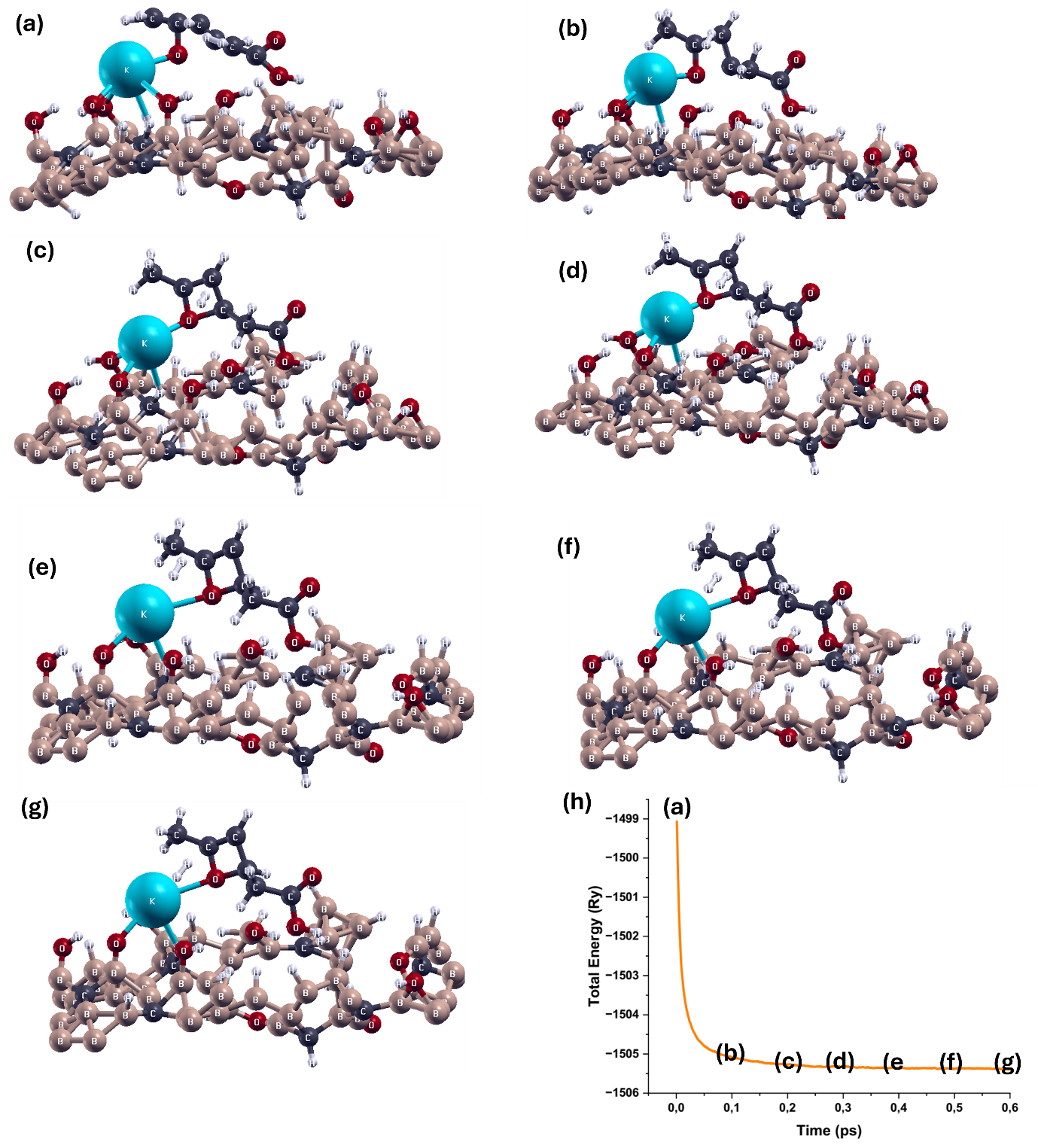
**

**Figure S21:** (a-g) MD simulations showing the interaction of LA with BH(6:1) surface at 250K; (h) Stability curve for the MD calculation s (energy vs time).


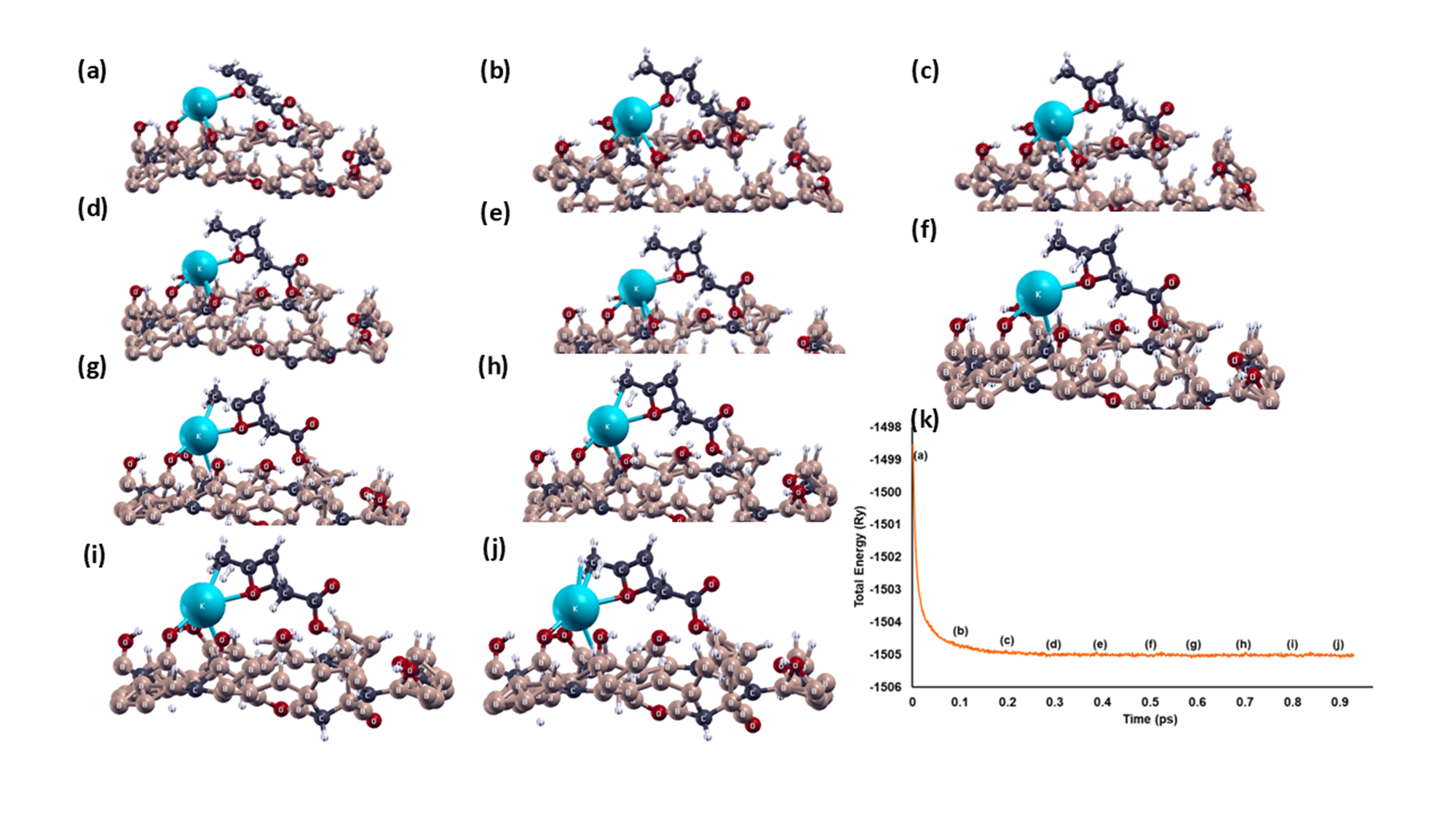


**Figure S22:** MD simulations showing the interaction of LA with BH(6:1) surface at 300K; Fig.(a-j) shows the progression of MD simulations; (k) Stability curve for the MD calculations (energy vs time).


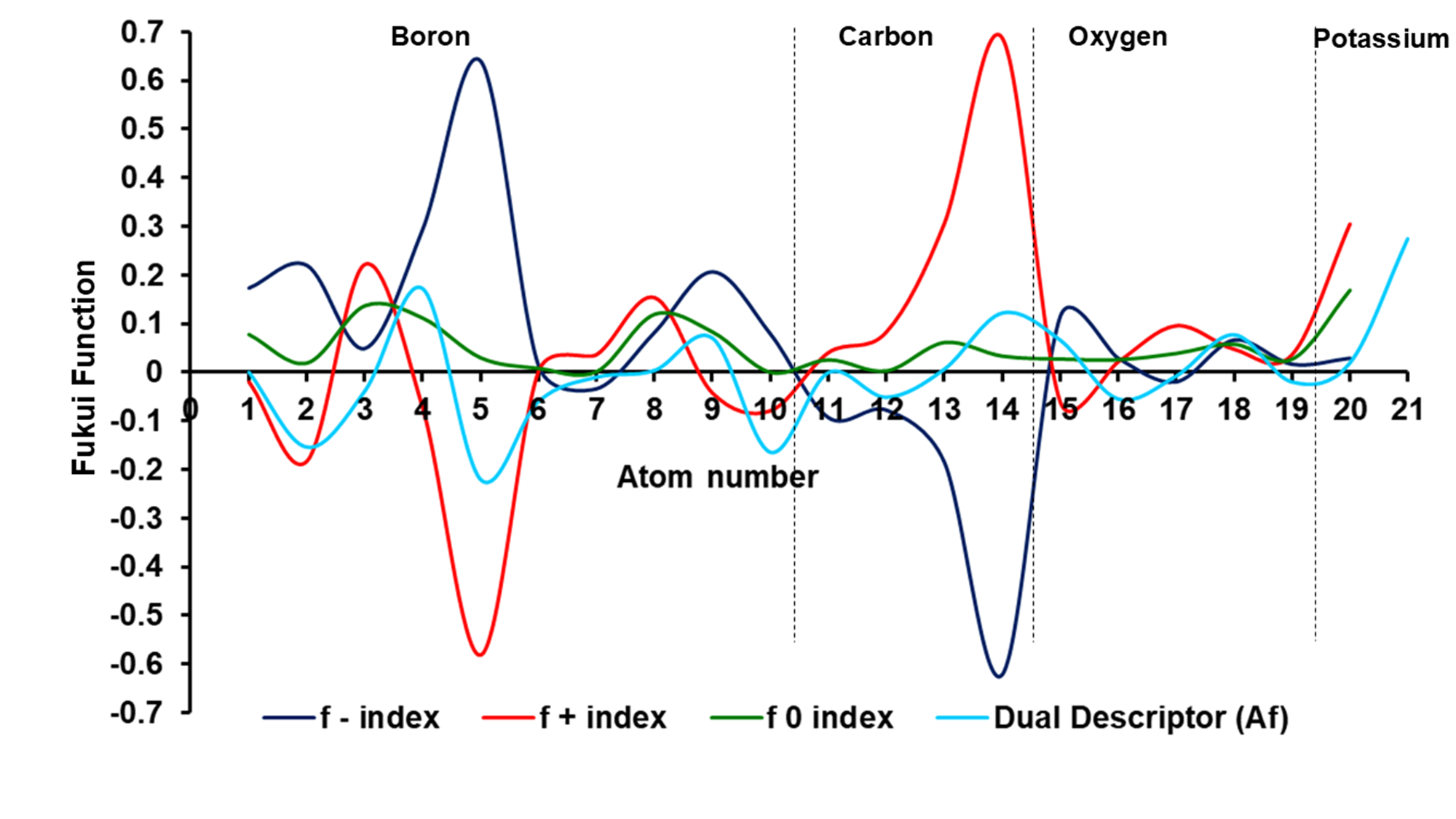
**Figure S23:** FUKUI function analysis of BH (6:1).

**References**

1. P. Kumar, V. C. Srivastava, Revealing Activity of Symmetrical and Asymmetrical Ag_2_O‐MO_x_ (M = Cu, Fe, Zn) Catalysts via DFT Calculations for Direct Propylene Epoxidation. *Adv Theory Simul.* **2025**, 8, 2400716.

2. P. Kumar, V. C. Srivastava, Ethane and Propane Dehydrogenation on Small Platinum Clusters Supported on Silica: An Ab Initio Molecular Dynamics and DFT Study. *Chempluschem*, **2024**, 89, e202300347.

3. P. Giannozzi, S. Baroni, N. Bonini, M. Calandra, R. Car, C. Cavazzoni, D. Ceresoli, G. L. Chiarotti, M. Cococcioni, I. Dabo, A. Dal Corso, S. de Gironcoli, S. Fabris, G. Fratesi, R. Gebauer, U. Gerstmann, C. Gougoussis, A. Kokalj, M. Lazzeri, L. Martin-Samos, N. Marzari, F. Mauri, R. Mazzarello, S. Paolini, A. Pasquarello, L. Paulatto, C. Sbraccia, S. Scandolo, G. Sclauzero, A. P. Seitsonen, A. Smogunov, P. Umari, R. M. Wentzcovitch, QUANTUM ESPRESSO: a modular and open-source software project for quantum simulations of materials. *Journal of Physics: Condensed Matter*., **2009**, 21, 395502.

4. J. P. Perdew, J. A. Chevary, S. H. Vosko, K. A. Jackson, M. R. Pederson, D. J. Singh, and C. FiolhaisErratum: Atoms, molecules, solids, and surfaces: Applications of the generalized gradient approximation for exchange and correlation. *Phys. Rev. B*, **1993**, 48, 4978.

5. K. B. and M. E. John P. Perdew, Generalized Gradient Approximation Made Simple. *Phys. Rev. Lett.* **1997**, 78, 1396.

6. P. E. Blöchl, Projector augmented-wave method. *Phys. Rev. B.,* **1994**, 50, 17953.

7. D. J. G. Kresse, From ultrasoft pseudopotentials to the projector augmented-wave method. *Phys. Rev. B* 1999, 59,1758.

8. K. Momma and F. Izumi, VESTA: a three-dimensional visualization system for electronic and structural analysis. *J Appl Crystallogr*, **2008**, 41, 653–658.

9. A Kokalj, XCrySDen--a new program for displaying crystalline structures and electron densities. *J Mol Graph Model*, **1999**, 17, 176–179.

10. R. M. Hanson, Jmol – a paradigm shift in crystallographic visualization. *J Appl Crystallogr*, **2010**, 43, 1250–1260.

11. N.K. Singh, P. Kumar, A. Yadhav, V. K. Srivastava, Multi-doped borophene catalysts with engineered defects for CO2 reduction: A DFT study. *J Colloid Interface Sci.,* **2024**, 654, 895–905.

12. J. Sánchez-Márquez, D. Zorrilla, A. Sánchez-Coronilla, D.M. de los Santos, J. Navas C. Fernández-Lorenzo, R. J. Alcántara, Martín-Calleja,Introducing “UCA-FUKUI” software: reactivity-index calculations. *Journal of molecular modeling*, **2014,** *20*(11), p.2492.
